# Patient outcomes associated with post-tuberculosis lung damage in Malawi: a prospective cohort study

**DOI:** 10.1136/thoraxjnl-2019-213808

**Published:** 2020-02-26

**Authors:** Jamilah Meghji, Maia Lesosky, Elizabeth Joekes, Peter Banda, Jamie Rylance, Stephen Gordon, Joseph Jacob, Harmien Zonderland, Peter MacPherson, Elizabeth L Corbett, Kevin Mortimer, Stephen Bertel Squire

**Affiliations:** 1 Department of Clinical Sciences, Liverpool School of Tropical Medicine, Liverpool, UK; 2 Malawi-Liverpool-Wellcome Clinical Research Programme, Blantyre, Malawi; 3 Division of Epidemiology and Biostatistics, University of Cape Town, Rondebosch, Western Cape, South Africa; 4 Department of Radiology, Royal Liverpool University Hospital, Liverpool, UK; 5 Department of Medicine, Queen Elizabeth Central Hospital, Blantyre, Southern Region, Malawi; 6 Centre for Medical Imaging and Computing, University College London, London, UK; 7 Department of Respiratory Medicine, University College London, London, UK; 8 Department of Radiology and Nuclear Medicine, Amsterdam University Medical Centres, Amsterdam, Noord-Holland, The Netherlands; 9 Clinical Research Department, London School of Hygiene and Tropical Medicine, London, UK; 10 Department of Infectious and Tropical Diseases, London School of Hygiene and Tropical Medicine, London, UK

**Keywords:** tuberculosis, bronchiectasis, clinical epidemiology, respiratory infection

## Abstract

**Background:**

Post-tuberculosis lung damage (PTLD) is a recognised consequence of pulmonary TB (pTB). However, little is known about its prevalence, patterns and associated outcomes, especially in sub-Saharan Africa and HIV-positive adults.

**Methods:**

Adult (≥15 years) survivors of a first episode of pTB in Blantyre, Malawi, completed the St George’s Respiratory Questionnaire, 6-minute walk test, spirometry and high-resolution CT (HRCT) chest imaging at TB treatment completion. Symptom, spirometry, health seeking, TB-retreatment and mortality data were collected prospectively to 1 year. Risk factors for persistent symptoms, pulmonary function decline and respiratory-related health-seeking were identified through multivariable regression modelling.

**Results:**

Between February 2016 and April 2017, 405 participants were recruited. Median age was 35 years (IQR: 28 to 41), 77.3% (313/405) had had microbiologically proven pTB, and 60.3% (244/403) were HIV-positive. At pTB treatment completion, 60.7% (246/405) reported respiratory symptoms, 34.2% (125/365) had abnormal spirometry, 44.2% (170/385) had bronchiectasis ≥1 lobe and 9.4% (36/385) had ≥1 destroyed lobe on HRCT imaging. At 1 year, 30.7% (113/368) reported respiratory symptoms, 19.3% (59/305) and 14.1% (43/305) of patients had experienced declines in FEV_1_ or FVC of ≥100 mL, 16.3% (62/380) had reported ≥1 acute respiratory event and 12.2% (45/368) had symptoms affecting their ability to work.

**Conclusions:**

PTLD is a common and under-recognised consequence of pTB that is disabling for patients and associated with adverse outcomes beyond pTB treatment completion. Increased efforts to prevent PTLD and guidelines for management of established disease are urgently needed. Low-cost clinical interventions to improve patient outcomes must be evaluated.

**Key messages**

**What is the key question?**

- What is the burden of post-tuberculosis lung damage among adults successfully completing pulmonary tuberculosis (pTB) treatment in urban Malawi, and what are the associated patient outcomes?

**What is the bottom line?**

- Pulmonary TB-survivors have a high burden of post-TB lung damage, which is largely undiagnosed within existing TB management pathways, and is associated with adverse patient outcomes including accelerated lung function decline, ongoing respiratory-related health seeking, persistent chest symptoms and difficulty working in the year after treatment completion.

**Why read on?**

- This work provides a detailed description of post-TB lung damage and associated outcomes among both HIV-positive and negative adults successfully completing pTB treatment in Malawi, and suggests research priorities for the prevention and management of disease.

## Introduction

An estimated 10.0 million incident cases of tuberculosis disease occurred globally in 2018, one-quarter of which were in Africa, where 29% of patients are HIV co-infected.^1^ TB mortality is falling, and 85% of people treated for a first episode of TB now survive with treatment success (cure or completion).^1^

Post-tuberculosis lung damage (PTLD) is a recognised consequence of pulmonary TB (pTB) disease: adult pTB-survivors have two-to-four-fold higher odds of persistently abnormal spirometry (airway obstruction and restriction) compared with those without previous TB disease,^2–4^ with parenchymal and airway abnormalities seen on imaging,^5 6^ and associated respiratory symptoms and reduced quality of life.^7–10^ However, there are few estimates of the burden of disease at pTB treatment completion, and few prospective data on the medium or long-term consequences of PTLD or risk factors for adverse patient outcomes. Data are particularly scarce for adults in low-income settings with HIV co-infection.^5^ There remain no standardised guidelines for the diagnosis and management of PTLD.^11^

Cohort studies from resource-rich settings suggest a correlation between the severity of chronic lung diseases and accelerated spirometry decline, hospital admissions and increased mortality.^12–14^ We hypothesise that adults with PTLD in low-income settings could experience similar—or more severe—adverse outcomes.

In this prospective cohort study in Malawi—one of the poorest countries in the world—we investigated the prevalence and pattern of residual lung damage at TB treatment completion using gold-standard respiratory investigations including high-resolution CT (HRCT) imaging and spirometry, with findings disaggregated by HIV-status. The evolution of pathology in the year following treatment completion is described and predictors of adverse outcomes identified.

## Methods

HIV-positive and HIV-negative adults expected to complete treatment for pTB at nine health centres in urban Blantyre, Malawi, between February 2016 and April 2017 were prospectively identified using the Malawi National Treatment Programme (NTP) registry. These individuals were screened by the study team at their monthly medication collection visits, with multiple opportunities available to identify each individual, and formal recruitment was completed at the end of treatment. Inclusion criteria were: age ≥15 years, residence in urban Blantyre, treatment for a first episode of pTB with cure or completion as defined by the NTP. Those with persistent symptoms at treatment completion underwent additional screening with sputum smear and TB culture and were excluded if either were positive. We excluded patients treated as multidrug resistant disease (MDR-TB prevalence among new TB cases in Malawi: 0.75%).^15^ All participants provided written informed consent.

Study visits were conducted at the central hospital within 1 month of pTB treatment completion, and at 6 months (home visit) and 12 months (hospital visit) after treatment end. Participants completed: questionnaires (demographics, respiratory exposures, socioeconomic data), the St George’s Respiratory Questionnaire (SGRQ); 6-minute walk test; pre-bronchodilator and post-bronchodilator spirometry; blood tests (full blood count, CD4 cell count, Aspergillus IgG). Imaging included chest radiography (CXR) at baseline and 12 months, and non-contrast HRCT chest imaging at baseline. Questionnaires and spirometry were repeated at all visits, and data on health seeking, TB retreatment and all-cause mortality were determined from participant-held health records. Details of existing cardiorespiratory diagnoses were obtained from health records, and TB microbiology at pTB diagnosis from NTP registers.

Questionnaires were conducted in the local language, Chichewa. HIV testing was offered to participants of unknown serostatus, and those who had tested over 1 month before recruitment (Serial testing with Determine 1/2; Alere, USA / Uni-Gold; Recombigen HIV, Trinity Biotech, Ireland). Plasma anti-Aspergillus fumigatus IgG was measured by ELISA (Bordier Affinity Products), using a cut-off index >1.0 for a positive result. Six-minute walk tests and spirometry (EasyOne, ndd Medical Technologies) were conducted to American Thoracic Society standards.^16 17^ HRCT imaging was performed using a prespecified protocol (online supplementary appendix 1). Participants underwent protocol-driven clinical review for investigation results requiring urgent intervention but attended routine clinical services for all other illness episodes.

10.1136/thoraxjnl-2019-213808.supp1 Supplementary data

### Quality control and data interpretation

Details of spirometry and imaging acquisition, quality control and interpretation are given in the online data supplement. Briefly, only spirometry data meeting the Burden of Obstructive Lung Disease (BOLD) study quality control standards were included in analyses (online supplementary appendix 2). Data were standardised using the Global Lung Initiative 2012 African-American reference ranges.^18^ Patterns of abnormality (obstruction: FEV_1_/FVC ratio <lower limit of normal (LLN); low FVC: FEV_1_/FVC ratio ≥LLN and FVC <LLN; normal: FEV_1_/FVC ratio ≥LLN and FVC ≥LLN) and reversibility (>200 mL and >12% increase in absolute FEV_1_ or FVC following bronchodilator) were described.^19^ HRCT images were independently read by two consultant radiologists with consensus review of discrepant findings (online supplementary appendix 3). The extent and severity of Fleischner-defined airway, parenchymal and pleural pathologies were recorded.^20^ Lobes where ≥90% of parenchyma was replaced by banding, atelectasis or cavities/cystic airspaces were classified as ‘destroyed’. Agreement between readers was measured using intraclass correlation coefficients and kappa scores.

### Statistical methods

A sample size of 400 allowed us to estimate the prevalence of PTLD with a margin of error less than 5% with 95% confidence (online supplementary appendix 4). We described the burden of respiratory pathology using clinical, spirometry and imaging parameters, stratified by HIV-status. X^2^, Student’s t-test, Fisher’s exact or Wilcoxon rank-sum tests were used to compare between groups. We compared the age-stratified prevalence of abnormal spirometry to recent community-based data from urban Blantyre in sensitivity analyses.^21^ Exploratory analyses were conducted to determine the relationship between symptoms, and spirometry and imaging parameters.

We determined the proportion of participants experiencing prespecified adverse outcomes over 1 year including: accelerated decline in FEV_1_ and FVC (loss ≥100 mL), chronic respiratory symptoms at 1 year (cough, breathlessness, sputum or wheeze ≥few days/month), TB-retreatment and all-cause mortality. We recorded the number of acute respiratory events, defined as ‘an unscheduled visit to healthcare provider, either outpatient or inpatient, due to a respiratory complaint (cough, breathlessness, sputum, wheeze, chest pain)’.

We used linear mixed effects and logistic models to estimate predictors of FEV_1_ and FVC over time and the prevalence of chronic respiratory symptoms and respiratory events by 1 year. Models were built using a prespecified set of covariates. Fixed effects and variance components were reported for linear mixed effects models, and coefficient estimates or ORs with 95% CIs otherwise. Separate outcome models were constructed using FEV_1_ and FVC as predictors, due to colinearity. Complete case analyses were performed in Stata 15 (StataCorp).

## Results

**Figure 1:**
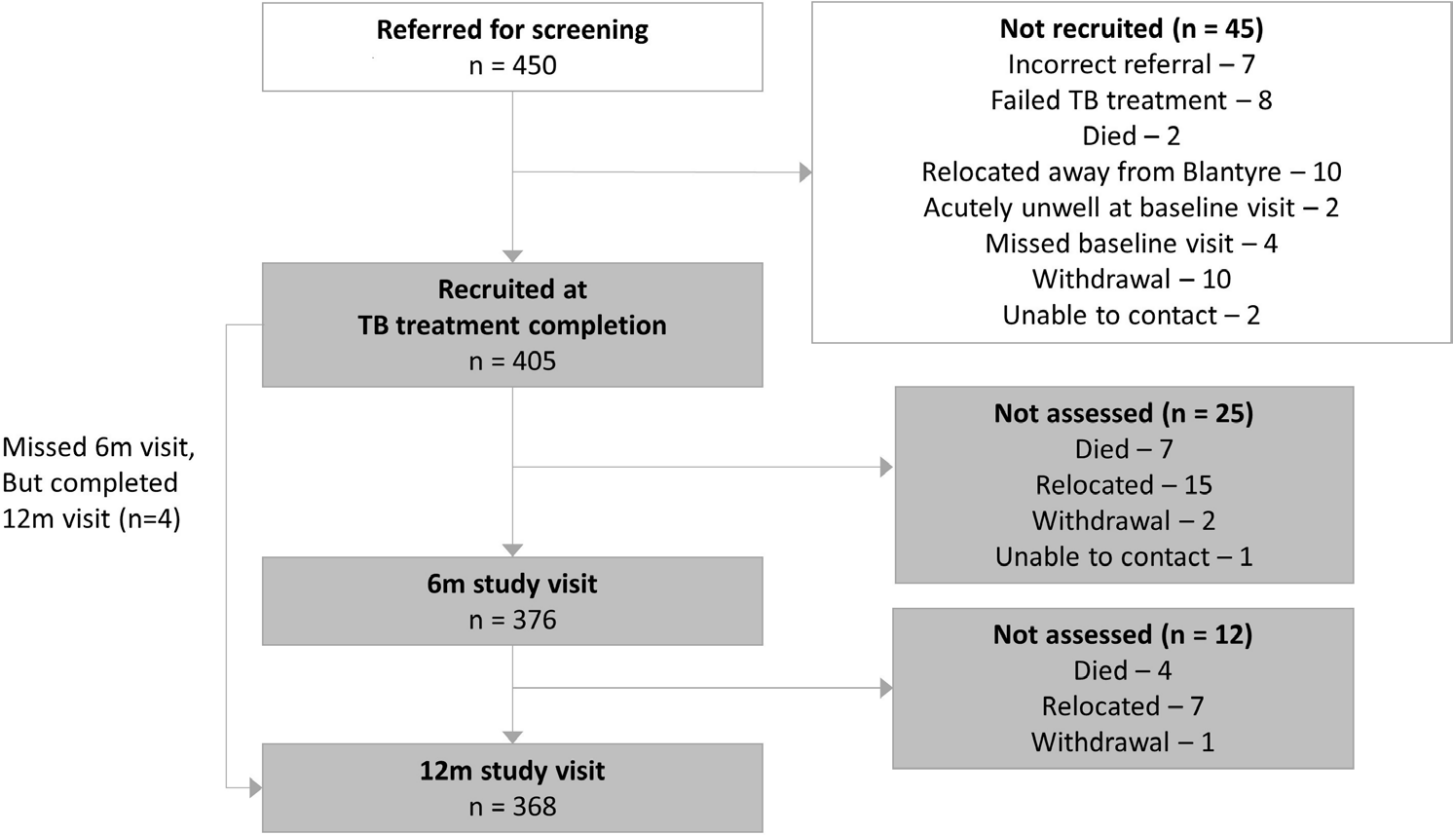
Participant flow diagram.

Four hundred and fifty pulmonary TB-survivors were screened for eligibility, of whom 405 met inclusion criteria at TB treatment completion (figure 1). 37/405 (9.1%) participants did not complete the final study visit: 22 relocated, 11 died, 3 withdrew and 1 was lost to follow-up. Participants not completing study procedures at 1 year had similar characteristics (age, sex, HIV status, TB microbiology, socioeconomic status, ever smoking/cannabis use) to those completing the study, but a higher prevalence of respiratory symptoms at baseline (75.7% (28/37) vs 59.2% (218/368), p=0.051).

### Participant characteristics

Median age was 35 years (IQR: 28 to 41), 67.9% (275/405) were male, and 77.3% (313/405) had microbiologically proven pTB disease (table 1). Among the 60.5% (244/403) who were HIV-positive, the majority were receiving antiretroviral therapy (ART) (91.8% (224/244)) and cotrimoxazole (90.2% (211/234)), with a median CD4 count of 229 cells/µL (IQR: 127 to 397) and median ART duration of 6.6 months (IQR: 5.5 to 25.6) by TB treatment completion. The median self-reported duration of illness prior to TB treatment initiation was 8.7 weeks (IQR: 4.0 to 13.0).

**Table 1:** Participant characteristics, stratified by HIV status*

Characteristic	Total	HIV-negative	HIV-positive	P value
	(n=405)	(n=159)	(n=244)	
Age (years) (median, IQR)	35 (28–41)	30 (24–37)	37 (32–42)	<0.001
Male sex	275 (67.9%)	115 (72.3%)	158 (64.8%)	0.112
Urban socioeconomic quintile (n=372)*				
Poorest	22 (5.9%)	10 (7.1%)	12 (5.2%)	0.638
Second poorest	85 (22.8%)	28 (19.9%)	57 (24.9%)	
Middle	95 (25.5%)	36 (25.5%)	58 (25.3%)	
Second most wealthy	114 (30.7%)	48 (34.0%)	66 (28.8%)	
Most wealthy	56 (15.1%)	19 (13.5%)	36 (15.7%)	
Maximum education level ≤primary school	154 (38.0%)	49 (30.8%)	104 (42.6%)	0.017
Intermittent difficulty procuring food for household	128 (31.6%)	47 (29.6%)	81 (33.2%)	0.443
Household dissaving incurred in past 1 year to cover illness costs†	298 (73.6%)	109 (68.6%)	189 (77.5%)	0.047
Monthly individual income ($US)‡	41.32 (11.02–96.42)	37.47 (5.51–82.64)	46.83 (16.53–99.17)	0.206
Baseline TB microbiology				
Smear positive	213 (52.6%)	118 (74.2%)	95 (38.9%)	<0.001
Xpert positive, rifampicin sensitive	100 (24.7%)	21 (13.2%)	78 (32.0%)	
Radiological diagnosis	54 (13.3%)	10 (6.3%)	43 (17.6%)	
Clinical diagnosis	38 (9.4%)	10 (6.3%)	28 (11.5%)	
Self-reported illness duration prior to TB treatment (weeks)	8.7 (4.3–13.0)	8.7 (4.3–13.0)	8.7 (4.3–17.4)	0.759
ART use, if HIV-positive (n=244)§			224 (91.8%)	
Duration on ART, if HIV-positive (months) (n=222)			6.6 (5.5–25.6)	
Prophylactic cotrimoxazole use, if HIV-positive (n=234)			211 (90.2%)	
CD4, if HIV-positive (cells/µL) (n=242)			229 (127–397)	
Ever smoker, cigarettes	120 (29.6%)	56 (35.2%)	62 (25.4%)	0.034
Pack years, among smokers (n=120)	2.7 (0.7–6.0)	2.1 (0.7–5.6)	2.9 (0.7–7.0)	0.465
Ever smoker, cannabis (n=362)*	54 (14.9%)	35 (25.4%)	19 (8.6%)	<0.001
Charcoal/wood as main fuel	384 (94.8%)	153 (96.2%)	229 (93.9%)	0.295

*Missing data: HIV status for n=2 (declined to test, included in ‘total’ column only), socioeconomic status (SES) for n=33 (unable to visit household to determine building materials), Cannabis use for n=43 (unbiased data collection error).

†Borrowing money, using savings, selling assets to cover costs due to illness, in past 1 year (during TB illness and treatment).

‡Income data collected in Malawi Kwacha, but standardised using exchange rate at study midpoint (1 US$: 726 MK, March 2017).

§97% (218/224) receiving Regimen 5a (tenofovir, lamivudine, efavirenz) at TB treatment completion.

ART, antiretroviral therapy.

Socioeconomic deprivation was common: 38.0% (154/405) were educated to primary school level only, 31.6% (128/405) reported intermittent food insecurity and 73.5% (298/405) had incurred dissaving (borrowing money, selling assets or using savings) to cover healthcare costs during TB illness or treatment.

Overall, 29.6% (285/405) of participants had ever-smoked, with a median of 2.7 pack-year (IQR: 0.7 to 6.0) exposure, and 13.3% (54/362) had ever-used cannabis. Use of biomass fuels for cooking/heating was reported by 94.8% (384/405). Only 2.2% (9/405) of the cohort had an established diagnosis of chronic lung disease (bronchitis n=4, asthma n=5) by treatment completion.

### Residual lung pathology at TB treatment completion

A majority of participants, 60.7% (246/405, 95% CI: 55.8% to 65.5%), reported one or more respiratory symptom at TB treatment completion (table 2). Median SGRQ total score was 8.8 (IQR: 1.3 to 23.4) and 40.0% (162/405, 95% CI: 35.2% to 45.0%) reported chest symptoms interfering with work. Median oxygen saturation was 98% (IQR: 97% to 99%). Hypoxaemia (<92%) was observed in 1.5% (6/405, 95% CI: 0.5% to 3.2%) at rest, and 3.8% (15/395, 95% CI: 2.1% to 6.2%) after the 6-minute walk test. A total of 17.5% (71/405) of participants were underweight (95% CI: 14.0% to 21.6% (body mass index <18.5 kg/m^2^)). Median haemoglobin was 13.7 g/dL (IQR: 12.3 to 15.1). Few participants (0.7% (3/405, 95% CI: 0.1% to 2.1%)) had positive *A.fumigatus* IgG serology (ELISA index >1.0).

**Table 2:** Clinical and respiratory parameters measured at TB treatment completion, 6-month and 12-month study visits

Parameter	TB treatment completion	6-month visit	12-month visit	P value:
	(n=405)	(n=376)	(n=368)	Baseline vs 12 months*
**Self-reported symptom prevalence (%,** **95%** ** CI** **)** **†**				
Breathlessness				
Never/only with chest infections	227 (56.0%, 51.1% to 60.9%)	283 (75.3%, 70.6% to 79.5%)	282 (76.6%, 72.0% to 80.9%)	<0.001
Few days per month	161 (39.8%, 35.0% to 44.7%)	79 (21.0%, 17.0% to 25.5%)	76 (20.7%, 16.6% to 25.2%)	
≥Several days per week	17 (4.2%, 2.5% to 6.6%)	14 (3.7%, 2.1% to 6.2%)	10 (2.7%, 1.3% to 4.9%)	
Cough				
Never/only with chest infections	259 (64.0%, 59.1% to 68.6%)	284 (75.5%, 70.9% to 79.8%)	307 (83.4%, 79.2% to 87.1%)	<0.001
Few days per month	135 (33.3%, 28.8% to 38.2%)	76 (20.2%, 16.3% to 24.6%)	54 (14.7%, 11.2% to 18.7%)	
≥Several days per week	11 (2.7%, 1.4% to 4.8%)	16 (4.3%, 2.5% to 6.8%)	7 (1.9%, 0.8% to 3.9%)	
Sputum production				
Never/only with chest infections	300 (74.1%, 69.5% to 78.3%)	300 (79.8%, 75.4% to 83.7%)	318 (86.4%, 82.5% to 89.7%)	<0.001
Few days per month	97 (23.9%, 19.9% to 28.4%)	70 (18.6%, 14.8% to 22.9%)	47 (12.8%, 9.5% to 16.6%)	
≥Several days per week	8 (2.0%, 0.9% to 3.9%)	6 (1.6%, 0.6% to 3.4%)	3 (0.8%, 0.2% to 2.4%)	
Wheeze				
Never/only with chest infections	372 (91.8%, 88.7% to 94.3%)	346 (92.0%, 88.8% to 94.6%)	352 (95.7%, 93.0% to 97.5%)	0.091
Few days per month	29 (7.2%, 4.8% to 10.1%)	28 (7.5%, 5.0% to 10.6%)	16 (4.3%, 2.5% to 7.0%)	
≥Several days per week	4 (1.0%, 0.3% to 2.5%)	2 (0.5%, 0.1% to 1.9%)	0	
Any respiratory symptom, ≥monthly	246 (60.7%, 55.8% to 65.5%)	138 (36.7%, 31.8% to 41.8%)	113 (30.7%, 26.0% to 35.7%)	<0.001
**Self-reported symptom impact (%,** **95%** ** CI** **)**				
Impact of chest on activities				
Does not stop any activities	200 (49.4%, 44.4% to 54.4%)	290 (77.1%, 72.5% to 81.3%)	295 (80.2%, 75.7% to 84.1%)	<0.001
Prevents one to two activities	165 (40.7%, 35.9% to 45.7%)	69 (18.4%, 14.6% to 22.6%)	57 (15.5%, 11.9% to 19.6%)	
Prevents most/all activities	40 (9.9%, 7.2% to 13.2%)	17 (4.5%, 2.7% to 7.1%)	16 (4.3%, 2.5% to 7.0%)	
Impact of chest on work				
Does not affect work	243 (60.0%, 55.0% to 64.8%)	309 (82.2%, 77.9% to 85.9%)	323 (87.8%, 84.0% to 90.9%)	<0.001
Interferes with/made me change work	148 (36.5%, 31.8% to 41.4%)	57 (15.2%, 11.7% to 19.2%)	38 (10.3%, 7.4% to 13.9%)	
Made me stop work	14 (3.5%, 1.9% to 5.7%)	10 (2.7%, 1.3% to 4.8%)	7 (1.9%, 0.8% to 3.9%)	
Breathless at rest/during personal care	2 (0.5%, 0.1% to 1.8%)	2 (0.5%, 0.1% to 1.9%)	2 (0.5%, 0.1% to 1.9%)	1.000
Walks slower than peers/stops for rest at own pace	108 (26.8%, 22.5% to 31.4%)	57 (15.2%, 11.7% to 19.2%)	64 (17.4%, 13.7% to 21.7%)	0.002
Breathless on hills	176 (43.7%, 38.8% to 48.7%)	82 (21.8%, 17.7% to 26.3%)	83 (22.6%, 18.4% to 27.2%)	<0.001
**Quality of life**				
Self-reported general health (%, 95% CI)				
Poor/fair	115 (28.4%, 24.1% to 33.1%)	54 (14.4%, 11.0% to 18.3%)	22 (6.0%, 3.8% to 8.9%)	<0.001
Good/excellent	290 (71.6%, 66.9% to 75.9%)	322 (85.6%, 81.7% to 89.0%)	346 (94.0%, 91.1% to 96.2%)	
SGRQ total score (median, IQR)	8.8 (1.3–23.4)	0.4 (0–10.6)	0.4 (0–6.9)	<0.001
SGRQ symptom score (median, IQR)	10.3 (2.7–23.1)	2.7 (0–13.7)	2.7 (0–13.7)	<0.001
SGRQ activity score (median, IQR)	11.2 (0–35.5)	0 (0–11.9)	0 (0–6.2)	<0.001
SGRQ impact score (median, IQR)	5.6 (0–15.5)	0 (0–5.7)	0 (0–3.7)	<0.001
**Clinical observations**				
BMI (kg/m^2^) (median, IQR)	20.5 (19.0–22.3)	21.0 (19.4–22.7)	21.1 (19.5–23.2)	<0.001
Oxygen saturations (%) (median, IQR)	98 (97–99)	98 (97–99)	98 (97–98)	<0.001
Hypoxaemia (sats<92%) (%, 95% CI)	6 (1.5%, 0.5% to 3.2%)	6 (1.6%, 0.6% to 3.4%)	4 (1.1%, 0.3% to 2.8%)	0.706
Respiratory rate (breaths/minute) (median, IQR)	18 (17–20)	19 (18–21)	20 (19–22)	<0.001
Heart rate (beats/minute) (median, IQR)	78 (68–89)	77 (68–86)	77 (67–86)	0.019
Pedal oedema (%, 95% CI)	7 (1.7%, 0.7% to 3.5%)	3 (0.8%, 0.2% to 2.3%)	3 (0.8%, 0.2% to 2.4%)	0.317
Palatal Kaposi sarcoma (n=368) (%, 95% CI)	8 (2.2%, 0.9% to 4.2%)	10 (2.7%, 1.3% to 4.8%)	1 (0.3%, 0.0% to 1.5%)	0.008
**Blood tests**				
Haemoglobin (g/dL) (median, IQR)	13.7 (12.3–15.1)			
Positive aspergillus IgG ELISA (%, 95% CI)	3 (0.7%, 0.2% to 2.1%)		2 (0.5%, 0.1% to 1.9%)	0.564
**6** ** min** **ute** **walk test (** **n** **=** **395** **/** **355** **)**				
Distance (m) (mean, SD)	568 m (79.7 m)		611.2 m (71.0 m)	<0.001
**Spirometry** **(** **n** **=** **365** **/** **341** **/** **336** **)** **‡**				
FEV_1_ z-score (mean, SD)	−1.06 (1.26)	−0.90 (1.25)	−0.88 (1.19)	<0.001
FVC z-score (mean, SD)	−0.91 (1.23)	−0.66 (1.19)	−0.61 (1.09)	<0.001
FEV_1_/FVC ratio z-score (mean, SD)	−0.38 (1.26)	−0.51 (1.28)	−0.54 (1.29)	<0.001
Pattern of spirometry (%, 95% CI)				
Obstruction (FEV1/FVC ratio <LLN)	52 (14.2%, 10.8% to 18.3%)	61 (17.9%, 14.0% to 22.4%)	60 (17.9%, 13.9% to 22.4%)	<0.001
Low FVC (FEV1/FVC ratio ≥LLN and FVC <LLN)	73 (20.0%, 16.0% to 24.5%)	45 (13.2%, 9.8% to 17.3%)	43 (12.8%, 9.4% to 16.8%)	
Normal (FEV1/FVC ratio ≥LLN and FVC ≥LLN)	240 (65.8%, 60.6% to 70.6%)	235 (68.9%, 63.7% to 73.8%)	233 (69.4%, 73.5% to 82.6%)	
**CXR findings (** **n** **=** **403** **/** **361** **)**				
% abnormal parenchyma (median (IQR), (range))	2.9 (0.4–9.2) (0–51.7)		2.1 (0–7.1) (0–70.8)	<0.001
Ring and tramline severity score (0–18) (median (IQR), (range))	1 (0–3) (0–13.5)		1 (0–2.5) (0–14.5)	0.1

*Pairwise comparisons between baseline and 12-month data using McNemar’s test for categorical variables, and Student’s t-test/Wilcoxon rank-sum for continuous variables.

†Symptom questions derived from SGRQ: Over the past 3 months I have (had shortness of breath / coughed / brought up sputum / had attacks of wheezing): not at all/only with chest infections / a few days a month / several days a week / most days a week; If you have tried to work in the past 3 months: my chest trouble does not affect my work / my chest trouble interferes with my work or made me change my work / my chest trouble made me stop work; Which of these statements best describes how your chest affects you: It does not stop me doing anything I would like to do / it stops me doing one to two things I would like to do / it stops me doing most of the things I would like to do / it stops me doing everything I would like to do.

‡BOLD standard data available for n=365/405 at baseline, n=341/376 at 6 months, and n=336/368 at 12 month study visits. Data age / sex / height standardised using GLI 2012 African-American reference ranges to generate z-scores.

BMI, body mass index; CXR, chest radiography; GLI-2012, Global Lung Initiative 2012; LLN, lower limit of normal; SGRQ, St George’s Respiratory Questionnaire.

BOLD standard post-bronchodilator spirometry data were available for 90.1% (365/405) of participants. Mean z-scores for the FEV_1_, FVC and FEV_1_/FVC ratio were negative (−1.06 (SD: 1.26), −0.91 (1.23), −0.38 (1.26), respectively). When classified into patterns, 20.0% (73/365, 95% CI: 16.0% to 24.4%) had a low FVC pattern and 14.2% (52/365, 95% CI: 10.8% to 18.3%) had airway obstruction. Among those with airway obstruction 9.6% (5/52, 95% CI: 3.2% to 21.0%) had reversibility. When the age-stratified prevalence of moderate-severe obstruction and low FVC patterns were compared with recent community-based data from urban Blantyre,^21^ the prevalence of both obstructive and low FVC patterns were higher in this post-TB cohort across age-strata (online supplementary appendix 5).

The prevalence of cough and exertional breathlessness were higher among HIV-negative compared with HIV-positive participants (45.9% (73/159) vs 29.5% (72/244), p=0.002 and 50.0% (79/158) vs 39.5% (96/243), p=0.038 respectively). Mean FEV_1_ and FVC z-scores were also significantly lower among HIV-negative participants (−1.27 (SD: 1.33) vs −0.94 (1.19), p=0.015 and −1.08 (1.29) vs −0.80 (1.18), p=0.037) (online supplementary appendix 6).

**Figure 2:**
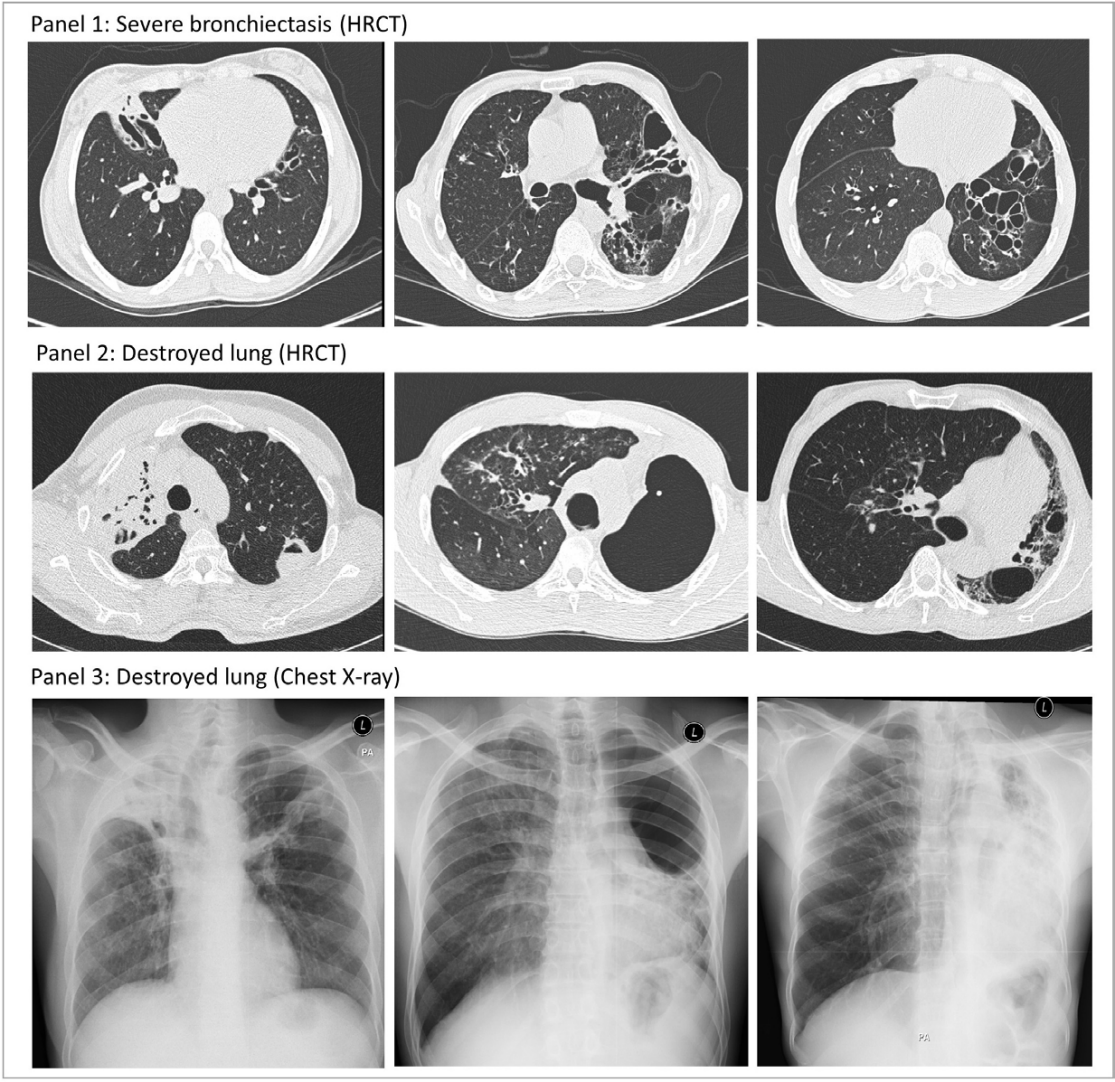
High resolution CT (HRCT) and chest x-ray imaging from participants with severe bronchiectasis (panel 1) or destroyed lung (panels 2 and 3, paired imaging from the same individuals), captured at TB-treatment completion. Bronchiectasis: Airway lumen diameter greater than accompanying pulmonary artery outer diameter, or airways visible <1 cm of lung periphery, or lack of normal airway tapering. Destroyed lung: ≥1 lung lobe in which ≥90% of parenchyma occupied by atelectasis or parenchymal banding, or destroyed by cavities/cystic airspaces.

**Figure 3:**
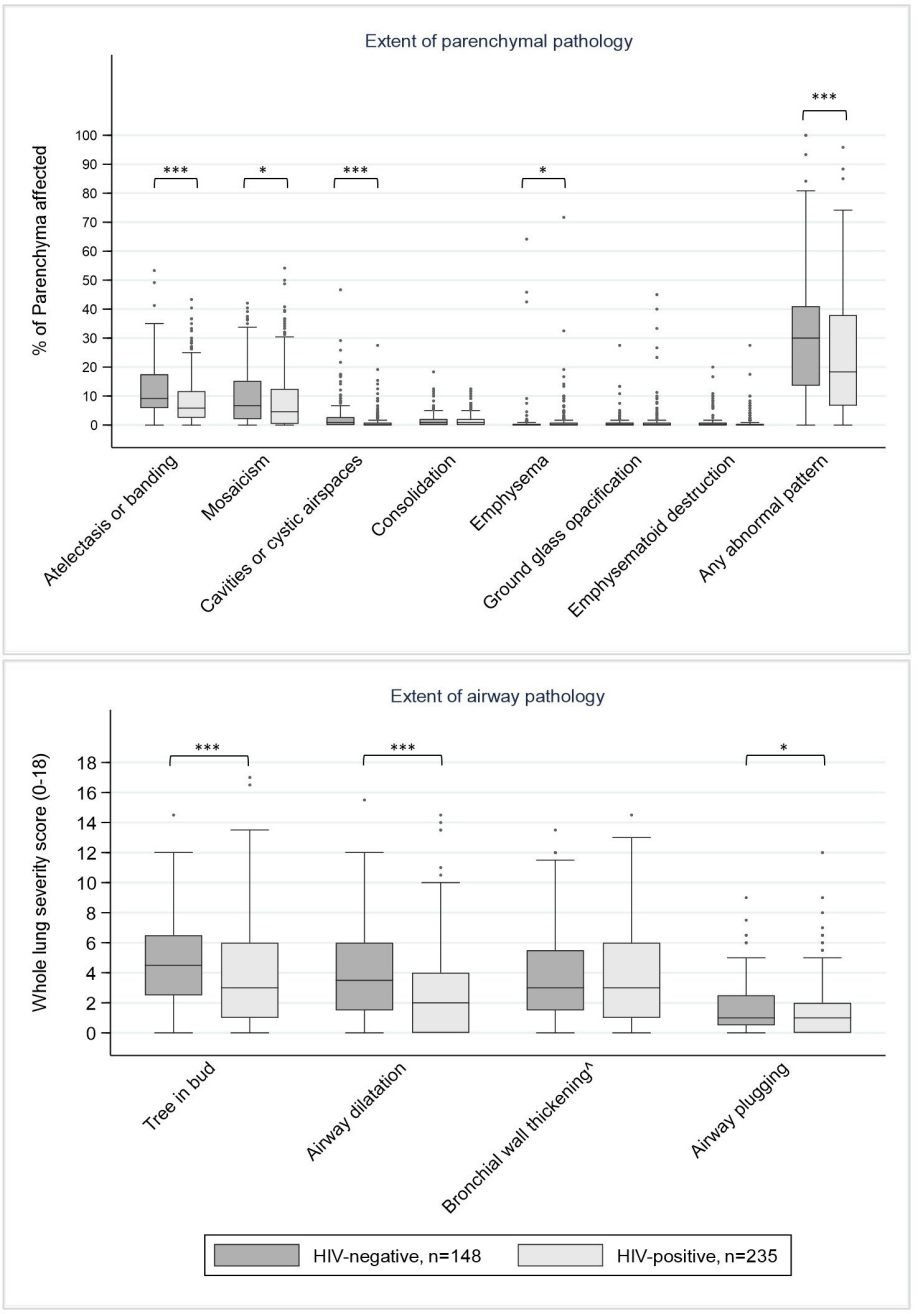
The extent of parenchymal and airway pathology seen on high-resolution CT imaging at TB treatment completion, stratified by HIV status (n=385). HRCT data missing for n=25: pregnancy (n=3), unable to travel to CT-scanner (n=6), missed appointments (n=8) and machine errors (n=3). ˆ Bronchial wall thickening: Not reported for those with lobar destruction preventing evaluation of bronchial wall thickness in ≥1 lung lobe (n=58). *p<0.05, **p<0.01, ***p<0.001.

In total 385 HRCT scans were completed, with 77.7% (299/385) within 2 months of TB treatment completion. Inter-reader agreement for the extent of Fleischner-defined parenchymal (intraclass correlation coefficient: 0.43 to 0.81) and airway abnormalities (kappa: 0.42 to 0.72) were good to excellent (online supplementary appendix 3).^20^ Moderate-to-severe bronchiectasis was seen in ≥1 lobe in 44.2% (170/385, 95% CI: 39.1% to 49.3%) of participants: 7.5% (29/385, 95% CI: 5.1% to 10.6%) had involvement of ≥3 lobes, and 12.7% (49/385, 95% CI: 9.6% to 16.5%) had cystic bronchiectasis. The median amount of abnormal parenchyma was 22.9% (IQR: 9.2% to 39.2%). Atelectasis and banding, and mosaicism were the most common patterns seen, and 9.4% (36/385, 95% CI: 6.6% to 12.7%) of participants had ≥1 destroyed lobe (figure 2). On average, the majority of airways and parenchymal pathologies were significantly more extensive in HIV-negative compared with HIV-positive participants (figure 3, online supplementary appendix 7). However residual consolidation, ground glass opacification and nodules were widespread (prevalence 69.4% (267/385, 95% CI: 64.5% to 73.9%), 36.6% (141/385, 95% CI: 31.8% to 41.7%) and 59.2% (228/385, 95% CI: 54.1% to 64.2%), respectively) with no significant difference by HIV-status.

Participants with weekly or monthly respiratory symptoms at TB treatment completion had lower FEV_1_ z-scores (−1.23 (SD: 1.28) vs −0.79 (1.19), p<0.001), lower FVC z-scores (−1.05 (SD: 1.25) vs −0.68 (1.17), p=0.013), more abnormal lung parenchyma (27.3% (95% CI: 10.0% to 42.9%) vs 18.3% (95% CI: 7.1% to 34.6%), p=0.002) and a higher proportion had ≥1 destroyed lung lobe on HRCT imaging (29/234 (12.4%, 95% CI:8.5% to 17.3%) vs 7/151 (4.6%, 95% CI: 1.9% to 9.3%), p=0.011) compared to those without regular symptoms. Those with regular cough had higher bronchiectasis severity scores than those without cough (3.0/18 (IQR: 1.0 to 5.5) vs 2.0/18 (0.5 to 4.5), p=0.014) (online supplementary appendix 8).

### Change in respiratory health over 1 year

On average, recovery was seen in respiratory health in the year after treatment completion: the prevalence of monthly symptoms declined (60.7% (246/405) to 30.7%(113/368), p<0.001), and average spirometry volumes increased (mean z-score change: FEV_1_ +0.20 (95% CI: 0.14 to 0.27) and FVC +0.33 (95% CI: 0.26 to 0.39)). However, by 1 year 12.2% (45/368, 95% CI: 9.1% to 16.0%) still had chest symptoms interfering with work, and mean spirometry z-scores remained negative (FEV_1_ −0.88 (SD: 1.19) and FVC −0.61 (SD: 1.09)) (table 2). In addition, 43.2% (159/368, 95% CI: 38.1% to 48.4%) of individuals had experienced a clinically significant deterioration in ≥1 respiratory parameter (symptoms, spirometry or imaging), including 19.3% (59/305, 95% CI: 15.1% to 24.2%) and 14.1% (43/305, 95% CI: 10.4% to 18.5%) of participants with a decline in FEV_1_ and FVC ≥100 mL (table 3).

**Table 3:** Proportion of participants experiencing clinically relevant improvement, deterioration or no change in respiratory parameters between baseline and 1 year study visits, among those completing both visits (n=368), with median size of change shown for continuous variables.

Parameter	Classification of change	Proportion of participants with each pattern of change (n, %)* Median (IQR) change observed		
		Improvement	No change	Deterioration
Self-reported general health	Change of ≥1 category in self-reported general health (poor, fair, good, excellent)	115 (31.3%)	229 (62.2%)	24 (6.5%)
BMI (kg/m^2^)	Change ≥1.46 kg/m2†	105 (28.5%) 2.7 (1.9 to 4.1)	244 (66.3%) 0.2 kg/m^2^ (−0.2 to 0.8)	19 (5.2%) −2.4 kg/m^2^ (−3.4 to −1.7)
SGRQ total score (n=366)	Change ≥4units‡	167 (45.6%) −16.1 points (−23.8 to −9.2)	153 (41.8%) −0.4 points (−1.8 to 0)	46 (12.6%) +11.5 points (7.0 to 25.9)
6 min walking distance (m) (n=348)	Change ≥26 m‡	201 (57.8%) 70 m (46 to 105 m)	102 (29.3%) 8 m (−4 to 15 m)	45 (12.9%) −49 m (−65 to −38 m)
Presence of monthly respiratory symptoms	Change between present / absent monthly symptoms	133 (36.1%)	207 (56.3%)	28 (7.6%)
FEV1 volume (L) (n=305)	Change ≥100 mL‡	133 (43.6%) 230 m (150 to 340)	113 (37.1%) 0 mL (−40 to 40)	59 (19.3%) −200 mL (−230 to −130)
FVC volume (L) (n=305)	Change ≥100 mL‡	164 (53.8%) 280 mL (190 to 400)	98 (32.1%) 30 mL (−40 to 60)	43 (14.1%) −160 mL (−220 to −120)
% abnormal parenchyma on CXR (n=359)	Change ≥4.68%†	58 (16.2%) −8.3% (−12.1 to −6.3)	283 (78.8%) 0% (−1.7 to 0.4)	18 (5.0%) +7.9% (6.3 to 13.3)
Ring and tramline score (0–18) on CXR (n=359)	Change ≥1.17 points†	79 (22.0%) −2.5 points (−4.0 to 2.0)	218 (60.7%) 0 points (−0.5 to 0)	62 (17.3%) 2.0 points (1.5 to 3.0)

*Pattern of change over 1 year period, classified using ‘Minimally important clinical difference (MCID)’ cut-offs for continuous variables, and change in response category for ordinal variables. Improvement: Increase ≥MCID or improvement by ≥1 category; No change: Remaining within +/-MCID of baseline reading, or remaining in same category; Deterioration: Reduction ≥MCID or deterioration by ≥1 category.

†No existing MCID agreed in literature: cut-off calculated by 0.5 x SD of baseline data.

‡MCID derived from COPD literature.^42^

BMI, body mass index; CXR, chest radiography; SGRQ, St George’s Respiratory Questionnaire.

Mixed effects models adjusted for spirometry at TB treatment completion found that FEV_1_ and FVC improved by an average of 70 mL (95% CI: 45 to 96 mL) and 131 mL (95% CI: 101 to 161 mL), respectively, over 1 year, with the greatest change seen in the first 6 months (online supplementary appendix 9). However, recovery was incomplete and the strongest predictor of spirometry at any time point was spirometry at TB treatment completion (accounting for >90% of total model variance). Accelerated FEV_1_ or FVC decline ≥100 mL was seen in participants across a wide range of baseline FEV_1_ and FVC measures (online supplementary appendix 10). The lowest spirometry volumes at 1 year were seen in those with the most extensive parenchymal pathology or bronchiectasis on HRCT, or the presence of symptoms at TB treatment completion.

### Chronic respiratory symptoms at 1 year

Respiratory symptoms were reported by 30.7% (113/368, 95% CI: 26.0% to 35.7%) at 1 year, with breathlessness being most common (table 2). On average, participants reporting respiratory symptoms at pTB treatment completion were significantly more likely to have symptoms 1 year later compared with those without baseline symptoms (OR 2.42 (95% CI: 1.37 to 4.27) and 2.45 (95% CI: 1.39 to 4.32) in models controlling for either baseline FEV_1_ or FVC). The odds of respiratory symptoms at 1 year were lower in HIV-positive compared with HIV-negative participants (OR 0.33 to 0.40 (95% CI: 0.18 to 0.98) across CD4 groups and models) (online supplementary appendix 11).

### Acute respiratory events over 1 year

Of participants who contributed 6 and 12 months of follow-up data, 25.0% (4/16, 95% CI: 7.3% to 52.4%) and 15.9% (58/364, 95% CI: 12.3% to 20.1%) experienced ≥1 acute respiratory event. The majority had one episode (77.4% (48/62)). Of the 70 unscheduled outpatient visits to local health centres or hospitals, 55.7% (39/70, 95% CI: 43.3% to 67.6%) were due to increased cough and 34.3% (24/70, 95% CI: 23.3% to 46.6%) were related to increased breathlessness. Antibiotics were prescribed in 80% of cases.

Participants with respiratory symptoms at treatment completion were more likely to report a respiratory event during follow-up (OR 2.6 (95% CI: 1.25 to 5.42)) compared with those without baseline symptoms. HIV-positive adults (12.9% (30/232, 95% CI: 8.9% to 17.9%)) had lower odds of a respiratory event compared with HIV-negative participants (21.9% (32/146, 95% CI: 15.5% to 29.5%)) in all models and at CD4 counts above and below 200 cells/µL (OR 0.33 to 0.43 (95% CI: 0.13 to 0.90)) (online supplementary appendix 12).

Controlling for baseline lung pathology the presence of more than one respiratory event during follow-up was negatively correlated with FEV_1_ and FVC volumes at 1 year. On average, those with ≥1 acute respiratory event had FEV_1_ and FVC volumes which were 82 mL (95% CI: 17 to 147 mL) and 122 mL (95% CI: 51 to 192 mL) lower at 1 year compared with those without events (online supplementary appendix 13).

### TB retreatment and mortality over 1 year

The TB symptom screen (≥1 of current cough, fevers, night sweats, weight loss, haemoptysis) was positive in 18.9% (71/376, 95% CI: 15.1% to 23.2%) and 10.6% (39/368, 95% CI: 7.6% to 14.2%) at the 6-month and 12-month visits, with current cough as the most common symptom reported (95.8% (68/71, 95% CI: 88.1% to 99.1%) and 92.3% (36/39, 95% CI: 79.1% to 98.4%) at 6 and 12 months). Sputum was obtained at 100/110 of these visits, and only 4% (4/100) had *Mycobacterium tuberculosis* (MTB) on culture. Three non-tuberculous mycobacteria isolates were cultured but repeat samples were negative. TB-retreatment was initiated in 3.7% (15/404) of participants during follow-up: two culture positive, five smear positive, four Xpert MTB/RIF positive, three radiological diagnosis, one unknown. 2.7% (11/404) of participants died, of whom 45.5% (5/11) had been initiated on TB retreatment and 90.9% (10/11) were HIV-positive.

## Discussion

This study used gold-standard measurement approaches to show that among a prospectively recruited, unselected cohort of pTB-survivors in a low income, high TB and HIV prevalence setting, the burden of PTLD is high: after a single episode of successfully treated pTB disease, one-third of patients have abnormal spirometry, over 40% have bronchiectasis and almost 10% have lobar destruction. This pathology is largely undiagnosed within existing TB management pathways, but is meaningful for patient outcomes, with accelerated lung function decline, ongoing respiratory-related health seeking, persistent chest symptoms and symptoms impairing work seen in 12% to 31% of patients in the year after treatment completion. Patterns of PTLD were heterogeneous, and although less severe among HIV-positive compared with HIV-negative patients, the burden of disease was marked in both groups.

The finding of a high burden of post-TB lung damage is consistent with previous literature which suggests two-to-four-fold increased odds of airway obstruction and restriction among those who have previously had pTB disease compared with those who have not,^2–4^ and ongoing airway and parenchymal imaging abnormalities following treatment success.^5 6^ Together, these data indicate a high population burden of respiratory pathology resulting from pTB disease.

To our knowledge, this is the first study to track change in lung function in an unselected patient cohort prospectively from pTB treatment completion. On average, FEV_1_ and FVC values for the cohort improved over time. Recovery was incomplete, but most marked in the first 6 months. This pattern of partial recovery is consistent with previous models,^22^ and is in keeping with the parenchymal destruction and airway dilatation seen on HRCT imaging which are unlikely to fully resolve.^23 24^ However, heterogeneity of lung function outcomes was observed between individuals within the cohort: while most experienced recovery, up to a fifth had a clinically meaningful decline in lung function over time. These patients are of particular concern given the known associations between reduced FEV_1_ and FVC, and increased mortality.^25–27^ In keeping with data from other chronic lung diseases, our analyses suggest that acute respiratory events in the year following treatment completion may drive worsening spirometry.^12 13^

Our data show that PTLD is relevant to patients’ lives and livelihoods. Chest symptoms interfering with work were reported by 46% at pTB treatment completion and 12% 1 year later. The high costs incurred by TB patients during diagnosis and disease treatment are well recognised and have been associated with adverse treatment outcomes.^28 29^ However, our findings suggest that post-TB morbidity may cause ongoing income losses even beyond treatment completion. These ongoing costs are not routinely included in calculations of the economic impact of TB disease, nor is the need to address them yet prioritised within the WHO ‘End-TB’ agenda, which aims to mitigate TB-related patient costs.^30^

Almost one-third of patients reported ongoing respiratory symptoms at 1 year. Chronic cough is stigmatising in high TB and HIV burden settings,^31^ and in this study also led to the WHO TB-symptom screening tool remaining positive for many patients, some months after their initial disease episode. Although pTB-survivors are at high risk of recurrent TB disease,^32^ empirical TB retreatment of pTB-survivors based on chronic symptoms is also widespread.^33^ In this study the majority of those reporting chronic cough during follow-up did not have microbiological evidence of pTB when retested, were not started on TB retreatment, and did not die, highlighting challenges with TB diagnosis in the post-TB population.

HIV-positive adults had less extensive PTLD compared with HIV-negative adults, despite similar self-reported illness duration prior to TB treatment initiation. HIV-TB co-infected adults with low CD4 counts have been shown to have less extensive CXR changes at TB diagnosis, due to impaired localised cellular immune responses to mycobacterial infection, and our data suggest that this translates into less extensive residual pathology at TB treatment completion.^34 35^ However, it is of note that although the burden of disease was lower among HIV-positive compared with HIV-negative adults, still moderate-to-severe bronchiectasis or abnormal spirometry was seen in a third of this group at pTB treatment completion. Most HIV-infected participants in this cohort were initiated on ART close to pTB treatment onset, with median CD4 count of only 229 cells/µL at TB treatment completion. Immune reconstitution on ART has been associated with lung inflammation and destruction during early treatment.^36 37^ Findings in this study may reflect a balance between the protective effect of low CD4 counts at TB diagnosis, and pro-inflammatory immune reconstitution with early initiation of ART.

Lastly, the heterogeneity of patterns of PTLD in this study was marked. Novel HRCT imaging findings included a high burden of mosaicism - this may reflect small airways disease but may also relate to pulmonary vascular disease, and echocardiography in this cohort would be of value.^38^ The high prevalence of consolidation, ground-glass and nodules even after 6 months of TB treatment was striking, but consistent with positron emission tomography-CT studies from South Africa which show ongoing metabolic activity in focal lung lesions of pTB-survivors, due either to persistent mycobacterial disease or a protracted host immune response to sterilised infection.^39^ The long-term relevance of this residual inflammation is not yet clear.

This is one of the first studies to prospectively investigate the nature and outcomes of post-TB lung damage, from the point of TB treatment completion, in a resource-poor setting in sub-Saharan Africa. The broad eligibility criteria mean that findings can likely be generalised to a wide spectrum of adults completing treatment in similar settings. Multiple respiratory parameters were measured to comprehensively describe pathology, with high standards of quality control and best-practice reporting, and outcome data were collected prospectively. The availability of CT imaging is limited in low- and middle-income countries, and its inclusion is therefore of particular value. Although the study was completed within an urban setting with a highly mobile population, loss to follow-up was under 10%.

As patients were not assessed prior to TB disease and no control group was included for comparison, the aetiology of lung pathology cannot be confirmed. The short follow-up duration of 1 year means that models of patient outcomes may have been underpowered and precluded investigation of risk factors for TB retreatment or mortality. Observed changes over time may be related to regression to the mean, test-retest variation or participant learning, but minimally important clinical difference cut-offs were used to allow for this. Study recruitment required attendance at a central hospital, and a selection bias away from those with severe disease may exist. Although this study was observational, participants likely received more medical advice than routinely available, and findings may be biased towards improved outcomes.

In summary, this study has found that PTLD is a common and under-recognised consequence of pTB that is disabling for patients and associated with adverse outcomes beyond pTB treatment completion. Our data highlight the importance of preventing PTLD: further investigation of host, environment and pathogen determinants of the nature and severity of PTLD, including HIV-specific factors, are required to identify upstream modifiable risk factors.^40^ Host directed therapies, and earlier TB diagnosis through active case finding and improved diagnostics may reduce lung damage, and we suggest that the burden of PTLD should be included as a secondary outcome in studies investigating the impact of these approaches.

Evidence-based guidelines for the management of those with established PTLD are lacking but urgently needed.^11^ It is not yet clear which interventions would be clinically and cost-effective at maximising health and preventing ongoing decline after pTB treatment completion, but our data suggest that appropriate management of respiratory exacerbations, and improved screening pathways for recurrent pTB disease should be prioritised. Other low-cost strategies including pulmonary rehabilitation and airway clearance exercises require evaluation, and health systems capable of providing long-term care to pTB-survivors will be needed to deliver these services.^41^ Ultimately, we suggest that renewed efforts by the global TB research and practice community to address the sequelae of TB disease, beyond treatment completion, will be required to improve long-term patient well-being.

## References

[1] World Health Organisation Global tuberculosis report, 2019. Geneva, Switzerland: World Health Organisation, 2019.

[2] Allwood BW, Myer L, Bateman ED A systematic review of the association between pulmonary tuberculosis and the development of chronic airflow obstruction in adults. Respiration 2013;86:76–85. 10.1159/000350917 23652030

[3] Byrne AL, Marais BJ, Mitnick CD, et al Tuberculosis and chronic respiratory disease: a systematic review. Int J Infect Dis 2015;32:138–46. 10.1016/j.ijid.2014.12.016 25809770

[4] Amaral AFS, Coton S, Kato B, et al Tuberculosis associates with both airflow obstruction and low lung function: BOLD results. Eur Respir J 2015;46:1104–12. 10.1183/13993003.02325-2014 26113680 PMC4594762

[5] Meghji J, Simpson H, Squire SB, et al A systematic review of the prevalence and pattern of imaging defined Post-TB lung disease. PLoS One 2016;11:e0161176 10.1371/journal.pone.0161176 27518438 PMC4982669

[6] Panda A, Bhalla AS, Sharma R, et al Correlation of chest computed tomography findings with dyspnea and lung functions in post-tubercular sequelae. Lung India 2016;33:592–9. 27890986 10.4103/0970-2113.192871 PMC5112814

[7] Báez-Saldaña R, López-Arteaga Y, Bizarrón-Muro A, et al A novel scoring system to measure radiographic abnormalities and related spirometric values in cured pulmonary tuberculosis. PLoS One 2013;8:e78926 10.1371/journal.pone.0078926 24223865 PMC3815252

[8] Banu Rekha VV, Ramaschandran R, Kuppu Rao KV, et al Assessment of long-term status of sputum positive pulmonary TB patients successfully treated with short course chemotherapy. Indian J Tuberc 2009;56:132–40. 20349754

[9] Chin AT, Rylance J, Makumbirofa S, et al Chronic lung disease in adult recurrent tuberculosis survivors in Zimbabwe: a cohort study. Int J Tuberc Lung Dis 2019;23:203–11. 10.5588/ijtld.18.0313 30808453 PMC6860915

[10] Pasipanodya JG, Miller TL, Vecino M, et al Using the St. George respiratory questionnaire to ascertain health quality in persons with treated pulmonary tuberculosis. Chest 2007;132:1591–8. 10.1378/chest.07-0755 17890471

[11] van Kampen SC, Wanner A, Edwards M, et al International research and guidelines on post-tuberculosis chronic lung disorders: a systematic scoping review. BMJ Glob Health 2018;3:e000745 10.1136/bmjgh-2018-000745 PMC6058174 30057796

[12] Chalmers JD, Goeminne P, Aliberti S, et al The bronchiectasis severity index. An international derivation and validation study. Am J Respir Crit Care Med 2014;189:576–85. 10.1164/rccm.201309-1575OC 24328736 PMC3977711

[13] Martínez-García MA, Soler-Cataluña J-J, Perpiñá-Tordera M, et al Factors associated with lung function decline in adult patients with stable non-cystic fibrosis bronchiectasis. Chest 2007;132:1565–72. 10.1378/chest.07-0490 17998359

[14] Jones RC, Donaldson GC, Chavannes NH, et al Derivation and validation of a composite index of severity in chronic obstructive pulmonary disease. Am J Respir Crit Care Med 2009;180:1189–95. 10.1164/rccm.200902-0271OC 19797160

[15] World Health Organisation TB country profile. Geneva, Switzerland: Malawi, 2016.

[16] Holland AE, Spruit MA, Troosters T, et al An official European respiratory Society/American thoracic Society technical standard: field walking tests in chronic respiratory disease. Eur Respir J 2014;44:1428–46. 10.1183/09031936.00150314 25359355

[17] Miller MR, Hankinson J, Brusasco V, et al Standardisation of spirometry. Eur Respir J 2005;26:319–38. 10.1183/09031936.05.00034805 16055882

[18] Quanjer PH, Stanojevic S, Cole TJ, et al Multi-Ethnic reference values for spirometry for the 3–95-yr age range: the global lung function 2012 equations. Eur Respir J 2012;40:1324–43. 10.1183/09031936.00080312 22743675 PMC3786581

[19] Pellegrino R et al Interpretative strategies for lung function tests. Eur Respir J 2005;26:948–68. 10.1183/09031936.05.00035205 16264058

[20] Hansell DM, Bankier AA, MacMahon H, et al Fleischner Society: glossary of terms for thoracic imaging. Radiology 2008;246:697–722. 10.1148/radiol.2462070712 18195376

[21] Meghji J, Nadeau G, Davis KJ, et al Noncommunicable lung disease in sub-Saharan Africa. A community-based cross-sectional study of adults in urban Malawi. Am J Respir Crit Care Med 2016;194:67–76. 10.1164/rccm.201509-1807OC 26788760 PMC4960629

[22] Hnizdo E, Singh T, Churchyard G Chronic pulmonary function impairment caused by initial and recurrent pulmonary tuberculosis following treatment. Thorax 2000;55:32–8. 10.1136/thorax.55.1.32 10607799 PMC1745584

[23] Ryu YJ, Lee JH, Chun E-M, et al Clinical outcomes and prognostic factors in patients with tuberculous destroyed lung. Int J Tuberc Lung Dis 2011;15:246–50. 21219689

[24] Rhee CK, Yoo KH, Lee JH, et al Clinical characteristics of patients with tuberculosis-destroyed lung. Int J Tuberc Lung Dis 2013;17:67–75. 10.5588/ijtld.12.0351 23232006

[25] Burney PGJ, Hooper R Forced vital capacity, airway obstruction and survival in a general population sample from the USA. Thorax 2011;66:49–54. 10.1136/thx.2010.147041 20980245

[26] Burney P, Jithoo A, Kato B, et al Chronic obstructive pulmonary disease mortality and prevalence: the associations with smoking and poverty—a BOLD analysis. Thorax 2014;69:465–73. 10.1136/thoraxjnl-2013-204460 24353008 PMC3995258

[27] Sin DD, Anthonisen NR, Soriano JB, et al Mortality in COPD: role of comorbidities. Eur Respir J 2006;28:1245–57. 10.1183/09031936.00133805 17138679

[28] Wingfield T, Boccia D, Tovar M, et al Defining catastrophic costs and comparing their importance for adverse tuberculosis outcome with multi-drug resistance: a prospective cohort study, Peru. PLoS Med 2014;11:e1001675 10.1371/journal.pmed.1001675 25025331 PMC4098993

[29] Tanimura T, Jaramillo E, Weil D, et al Financial burden for tuberculosis patients in low- and middle-income countries: a systematic review. Eur Respir J 2014;43:1763–75. 10.1183/09031936.00193413 24525439 PMC4040181

[30] World Health Organisation The end TB strategy. Geneva, Switzerland, 2015.

[31] Chikovore J, Hart G, Kumwenda M, et al Tb and HIV stigma compounded by threatened masculinity: implications for TB health-care seeking in Malawi. Int J Tuberc Lung Dis 2017;21:26–33. 10.5588/ijtld.16.0925 29025482

[32] Marx FM, Floyd S, Ayles H, et al High burden of prevalent tuberculosis among previously treated people in southern Africa suggests potential for targeted control interventions. Eur Respir J 2016;48:1227–30. 10.1183/13993003.00716-2016 27390274 PMC5512114

[33] Metcalfe JZ, Mason P, Mungofa S, et al Empiric tuberculosis treatment in retreatment patients in high HIV/tuberculosis-burden settings. Lancet Infect Dis 2014;14:794–5. 10.1016/S1473-3099(14)70879-5 PMC4317243 25164190

[34] Ravimohan S, Auld SC, Maenetje P, et al Lung injury on antiretroviral therapy in adults with human immunodeficiency Virus/Tuberculosis. Clinical Infectious Diseases 2019;163 10.1093/cid/ciz560 PMC7156779 31242296

[35] Chamie G, Luetkemeyer A, Walusimbi-Nanteza M, et al Significant variation in presentation of pulmonary tuberculosis across a high resolution of CD4 strata. Int J Tuberc Lung Dis 2010;14:1295–302. 20843421 PMC3033769

[36] Ravimohan S, Auld SC, Maenetje P, et al Lung injury on antiretroviral therapy in adults with HIV/TB. Clin Infect Dis 2019. 10.1093/cid/ciz560 PMC7156779 31242296

[37] Walker NF, Wilkinson KA, Meintjes G, et al Matrix degradation in human immunodeficiency virus type 1–Associated tuberculosis and tuberculosis immune reconstitution inflammatory syndrome: a prospective observational study. Clin Infect Dis 2017;65:121–32. 10.1093/cid/cix231 28475709 PMC5815569

[38] Kligerman SJ, Henry T, Lin CT, et al Methods of differentiation, and pitfalls. Radiographics: a review publication of the Radiological Society of North America, Inc 2015;35. 10.1148/rg.2015140308 26274445

[39] Malherbe ST, Shenai S, Ronacher K, et al Persisting positron emission tomography lesion activity and Mycobacterium tuberculosis mRNA after tuberculosis cure. Nat Med 2016;22:1094–100. 10.1038/nm.4177 27595324 PMC5053881

[40] Ravimohan S, Kornfeld H, Weissman D, et al Tuberculosis and lung damage: from epidemiology to pathophysiology. Eur Respir Rev 2018;27:170077 10.1183/16000617.0077-2017 29491034 PMC6019552

[41] Harries AD, Ade S, Burney P, et al Successfully treated but not fit for purpose: Paying attention to chronic lung impairment after TB treatment. Int J Tuberc Lung Dis 2016;20:1010–4. 10.5588/ijtld.16.0277 27393532

[42] Jones PW, Beeh KM, Chapman KR, et al Minimal clinically important differences in pharmacological trials. Am J Respir Crit Care Med 2014;189:250–5. 10.1164/rccm.201310-1863PP 24383418

