# Supplementary material for: Patient outcomes associated with post-tuberculosis lung damage in Malawi: a prospective cohort study
Source: Thorax. 2020 Feb 26;75(3):269–78. doi: 10.1136/thoraxjnl-2019-213808 (PMC7063395; doi:10.1136/thoraxjnl-2019-213808)
Supplement: Supplementary data [file thoraxjnl-2019-213808supp001.pdf]

Supplementary Materials

Manuscript title:

Patient outcomes associated with post-tuberculosis lung damage in Malawi: a prospective cohort study

Authors:

Jamilah Meghji, Maia Lesosky, Elizabeth Joekes, Peter Banda, Jamie Rylance, Stephen B Gordon, Joseph Jacob, Harmien Zonderland, Peter MacPherson, Elizabeth L Corbett, Kevin Mortimer, Stephen B Squire

Meghji J, *et al. Thorax* 2020; 75:1–10. doi: 10.1136/thoraxjnl-2019-213808

Appendix 1: CT scanning protocol

All participants were offered low-dose HRCT imaging at pTB treatment completion, except for those with contraindications: absence of informed consent, positive pregnancy test / self-reported current pregnancy, inability to travel to the imaging centre. Those with positive TB symptom screens (cough, weight loss, fever, night sweats, haemoptysis) at pTB-treatment completion were asked to submit sputum, and only booked for imaging if smear negative.

For logistical reasons, 2 imaging centres were used: Blantyre Adventist Hospital (BAH), Blantyre (Feb – Aug 2016) and Kamuzu Central Hospital (KCH), Lilongwe (Nov 2016 – May 2017). The same unenhanced low dose imaging protocol was used at both sites (Table E1). Imaging was performed supine in full inspiration, with coverage from apices to bases. Scans were reconstructed using a lung algorithm only, accepting that this would give limited diagnostic quality for soft tissue. Images were stored in DICOM format electronic files.

Table E1: Non contrast high resolution CT scanning protocol

Topogram

mA	kV	Scan time	Slice	Topogram length	Tube position	Direction	API	Kernel	Window	Tilt
50	120	5.3 sec	1.0 mm	512 mm	Top	Cranio-caudal (Supine)	Inspiration	T20s Standard	Topogram body	0.0°

Scan

Effective mAs	kV	Scan time	Delay	Slice	CTDL Vol	Pitch	Acquisition	Care dose
50 depending on patient size	120 depending on patient size	12.05	5 sec	Volume HRCT 1mm at 1mm intervals	3.90 mGy	1.15	Spiral scan	Care Dose 4D

Processing

Reconstuction	Image order	Reconstruction increment	Kernel	Window	Field of view	Centre X	Centre Y	3D
Axial	Cranial-caudal	1.0mm	B60f	Lung window	380	0 mm	0 mm	VRT & MIP as needed

Meghji J, *et al. Thorax* 2020; 75:1–10. doi: 10.1136/thoraxjnl-2019-213808

Appendix 2: Spirometry quality control procedures

Spirometry measurement

Pre- and post-bronchodilator spirometry were performed at each study visit – each participant performed up to eight attempts for each, until three high quality curves were obtained. Where consistently poor technique / difficulty performing the test was observed, participants were asked to return on another occasion for repeat testing. Salbutamol was administered via a spacer after the pre-bronchodilator tests, using two 100mcg doses from an MDI device. A minimum of 15 minutes was allowed between administration of salbutamol and repeat spirometry. Nose clips were found to function poorly in the study population, so participants were asked to pinch their noses during testing. Disposable spirettes were used and changed for each patient. Testing was performed in the seated position during baseline and 12-month visits, and at the 6-month visit if suitable seating was available. Participant age (years), standing height (mm), and weight (kg) were recorded contemporaneously for standardization of results. Spirometry data were stored electronically using EasyWare software.

Spirometry quality control

All pre- and post- spirometry attempts were reviewed by two readers (JM & LZ) independently, and graded for errors according to the CDC National Institute for Occupational Safety & Health (NIOSH) guidelines, and the BOLD study quality control procedures (Table 2).(1, 2) Tests were reviewed in chronological order in batches of 100, and grading was resolved by consensus discussion in the event of discrepancy. The 5% of usable spirometry readings with the longest Forced Expiratory Times (FET) values, and readings with the 1% highest and lowest FEV_1 and FVC values were manually reviewed, and patient results compared across study visits to ensure consistency and accuracy. Curves which

were clearly inconsistent with other data for a given patient, where a zero-flow error was suspected, or where there was suspicion of an error in study ID or test performance, were classified as invalid.

Table E2: Grading criteria for individual spirometry trials, with source of guidelines listed

Reason for rejection of spirometry curve	Abbreviation for error	Definition of error	Reference/ source
High PEFT	p (PEFT)	PEFT ≥150msecs	BOLD QC requirements, which relaxed the ndd cut off of 120ms
High BEV	b (BEV)	BEV ≥150ml<br>AND<br>BEV ≥5% of FVC	ATS criteria, NIOSH, BOLD QC requirements state that for a curve to be included BEV must be <5% or <150ml, whichever is greater.
Non-maximal effort	e (effort)	Marked lack of peak, indicating weak blast<br>OR<br>Markedly reduced peak compared to other curves, indicating poor filling of lungs at start of test	BOLD QC requirements<br>ATS criteria<br>NIOSH guidelines
Early termination of expiration	t (termination)	Insufficient expiratory phase on volume-time curve – duration of expiration for < 6 secs OR failure to reach plateau of ≥1 sec<br>OR<br>Sharp early drop to 0 on flow-volume curve	BOLD QC requirements<br>ATS criteria<br>NIOSH guidelines
Extra breath	x (extra)	Visible extra breath on flow-volume and or the volume-time curves	BOLD QC requirements<br>NIOSH guidelines
Glottis closure that influences measurement	g (glottis)	Abrupt flat line on volume-time curve, with sharp drop to 0 on flow-volume curve	BOLD QC requirements<br>ATS criteria<br>NIOSH guidelines
Leak	l (leak)	Descent of volume-time curve, after peak is reached, with 'back-track' of flow-volume curve at the end of expiration	BOLD QC requirements<br>ATS criteria<br>NIOSH guidelines
Obstructed mouthpiece	o (obstruction)	Artefact in the flow-volume and volume-time curves, felt to be significant enough to affect measurement	BOLD QC requirements<br>ATS criteria<br>NIOSH guidelines
Cough that affects measurement	c (cough)	Cough within 1st second which is likely to alter FEV_1, or a later cough which causes early termination.	BOLD QC requirements<br>ATS criteria<br>NIOSH guidelines
Zero flow error	z (zero)	Continuous rise of volume-time curve, with no plateau, and long tail on flow-volume curve, which is felt related to error rather than obstructive impairment	BOLD QC requirements<br>NIOSH guidelines

PEFT: Peak expiratory flow time; BEV: Back extrapolated volume; BOLD: Burden of Obstructive Lung Disease Study; ATS: American Thoracic Society; NIOSH: National Institute for Occupational Safety & Health, Centres for Disease Control & Prevention; QC: Quality Control.

Data were used for a given patient at a given study visit only if 2 usable curves with no errors were available, and if the differences between both the best/next-best FEV_1 and FVC readings between these curves was ≤200ml. Patients with spirometry which did not meet these criteria were defined as having 'missing' data for this study visit.

Meghji J, *et al. Thorax* 2020; 75:1–10. doi: 10.1136/thoraxjnl-2019-213808

Spirometry interpretation

Spirometry data meeting these standards were standardised for age, sex and height using the Global Lung Initiative 2012 (GLI-2012) African reference ranges.(3) Data were described using z-scores, and 5% lower limit of normal (LLN) cut-offs used to determine patterns of abnormality (obstruction: FEV_1/FVC ratio <LLN; low FVC: FEV_1/FVC ratio ≥LLN & FVC<LLN; normal: FEV_1/FVC ratio ≥LLN & FVC≥LLN). Reversibility was defined as a >200ml and >12% increase in absolute FEV_1 or FVC following bronchodilator.(4)

Meghji J, *et al. Thorax* 2020; 75:1–10. doi: 10.1136/thoraxjnl-2019-213808

Appendix 3: HRCT reporting procedures

Derivation of scoring tool

There exist no validated image scoring tools for the measurement of post-TB lung damage. A novel tool was therefore developed for use here (Table 3). This was informed by a systematic review of the existing literature on post-TB lung damage,(5) pictorial essays of chest imaging at various stages of PTB disease, and review of image scoring systems commonly used in bronchiectasis, COPD, and interstitial lung disease studies. It was developed by the study PI (JM) and two consultant radiologists: a consultant chest radiologist with predominantly UK specialist respiratory experience (JJ), and a UK-based ID radiologist with experience of TB related pathology and imaging in sSA (EJ). Particular attention was paid to the selection of variables used to measure bronchiectasis and airways pathology. Established radiological criteria from the Fleischner guidelines were used throughout,(6) except for the category of 'Emphysematoid destruction' which is a feature described in the TB and interstitial lung disease (ILD) literature only.(7) Joint scoring of a training set of CT images obtained from a previous study of patients receiving TB-retreatment in urban Blantyre was used to refine the scoring tool prior to use.

Image scoring

All HRCT images were independently scored by two consultant radiologists (EJ and JJ). Anonymised images were used, with no accompanying demographic or health related information, and images were provided to both radiologists in the same format and order over the course of the study. CTs were scored on a lobar basis. For scoring purposes the lingula was counted as a separate lobe using the level of the origin of the lingula bronchus to demarcate the boundary of the lingula from the left upper lobe. Scores were entered directly into a live reporting database.

Meghji J, *et al. Thorax* 2020; 75:1–10. doi: 10.1136/thoraxjnl-2019-213808

Data from the first 20 independently reported scans from within this study were openly reviewed by both primary readers together to consolidate training. Re-scoring of initial data for these first 20 scans was permitted following this review, but all subsequent images were independently reported with no comparison or changes allowed.

Table E3: Novel HRCT chest scoring tool, for post-tuberculosis lung damage

Variable	Definition	Lobar level scoring options
PARENCHYMAL VARIABLES	Scored at lobal level. Percentage of parenchyma affected by each pattern estimated to nearest 5%. Lobar percentages summed over whole lung to give total lung score /600.	
Parenchymal bands	Linear opacity 1-3mm thick, up to 5cm long. Usually extends to visceral pleura.<br>May be accompanied by anatomical distortion	% of parenchyma (to nearest 5%)
Atelectasis	Reduced lung volume which may be accompanied by displacement of fissures, bronchi, vessels.	
Consolidation	Homogeneous increase in lung parenchymal attenuation which obscures the margins of vessels and airway walls. An air bronchogram may be present.	
Ground glass opacification	Hazy increased lung opacity with preservation of bronchial and vascular margins	
Mosaicism	Specifically, the low attenuation component of a variable "mosaic" attenuation pattern within the lung	
Emphysema	Focal areas or regions of low attenuation usually without visible walls	
Emphysematoid destruction	Focal area of destruction/emphysematous change associated with features of healing TB, suggesting destruction of acini/small airways.	
Cavities / cystic airspaces	Gas filled structure, seen as a lucency or low-attenuation area within a pulmonary consolidation, a mass, or a nodule. Consolidation may have resolved leaving a thin wall only.	
Normal	Normal parenchyma, not affected by any of the pathological processes above	
BRONCHIECTASIS VARIABLES	Scored at lobar level. Lobar extent and severity scores summed across whole lung to give total score /18.	
Bronchiectasis<br><br>Airway lumen diameter greater than accompanying pulmonary artery outer diameter,<br>OR<br>Airways visible within 1 cm of the lung periphery,<br>OR<br>Lack of normal airway tapering	Extent<br>(Maximal score of 2 for middle lobes – 2 segments only)	0: Absent<br>1: ≤1 BP segment<br>2: 2 BP segments<br>3: ≥ 3 BP segments
	Pattern<br>(Nominal variable, scored only if bronchiectasis seen and 'extent' score >0)	1: Cystic ('Ballooned' outline, with diameter increasing towards periphery)<br>2: Cylindrical (Regular and straight outline, with abrupt termination)<br>3: Varicose (Irregular beaded bronchial outline with bulbous termination)
	Severity<br>Maximum degree of airway dilatation, to be measured by comparing diameter of airway lumen to diameter of adjacent vessel.<br>(Scored only if bronchiectasis seen and 'extent' score >0)	1: Trivial (bronchial lumen is <twice adjacent vessel diameter)<br>2: Bronchial lumen is 2-3 times adjacent vessel diameter<br>3: Bronchial lumen is >3 times adjacent vessel diameter
AIRWAY VARIABLES	Scored at lobar level. All variables independent of each other. Lobar scores summed across whole lung to give total score /18.	

Bronchial wall thickening	Thickening of bronchial walls	0: Absent<br>1: Mild<br>2: Moderate<br>3: Severe<br>Missing: Unable to assess
Airway plugging	Plugging seen in large airways	0: Absent<br>1: Mild<br>2: Moderate<br>3: Severe
Tree in bud	Centrilobular branching pattern in the peripheral airways, resembling a budding tree.	0: Absent<br>1: Mild<br>2: Moderate<br>3: Severe
CAVITY VARIABLES	Scored at lobar level. Independent of parenchymal scores. Lobar scores combined to give whole-lung data.	
Cavity / cystic airspace<br><br>Gas filled space, seen as a lucency or low-attenuation area within a pulmonary consolidation, a mass, or a nodule. Consolidation may have resolved leaving a thin wall only.	Extent	0: Absent<br>1: 1-2 cavities<br>2: 3-5 cavities<br>3: >5cavities
	Maximum size<br>(Scored only if bronchiectasis seen and 'extent' score >0)	Maximum diameter (mm)
	Mycetoma<br>Discrete mass of hyphae, within a cavity. May have air crescent sign. May have sponge like pattern with areas of calcification.<br>(Scored only if bronchiectasis seen and 'extent' score >0)	0: Absent<br>1: Present
OTHER LOBAR VARIABLES	Scored at lobar level, with scores combined to give whole-lung data	
Nodules	Rounded opacities, well or poorly-defined, >5mm, measuring up to 3cm in diameter	0: Absent<br>1: <5 nodules<br>2: ≥5 nodules<br>3: Miliary
WHOLE LUNG VARIABLES	Scored at level of hemithorax / whole lung	
Pleural effusion	Accumulation of fluid within pleural space	0: Absent<br>1: Present
Pleural thickening	Pleural thickening of ≥10mm	0: Absent<br>1: Present
Lymph nodes	Mediastinal / hilar lymph nodes ≥10mm diameter	0: Absent<br>1: Present

Lobar scores generated for each variable were summed across the whole lung, and these whole lung scores were then compared between readers. The Cohen's kappa score (binary variables), weighted Cohen's kappa score with quadratic weighting (ordinal variables with multiple categories), and intra-class correlation coefficients (continuous variables) were calculated to measure inter-reader agreement.

Consensus review of imaging reports

Consensus review was completed for the scans with the most discrepant scores for reported variables (Table 5), with cut-offs specified after review of the primary reporting data. For continuous variables, the most discrepant ~5% of scans for each variable were selected for review: assuming that the difference in scores between readers had a normal distribution, this rule identified scans with differences >2 standard deviations away from the mean difference for each variable, but allowed the absolute magnitude of discrepancy tolerated to vary between variables. Where a variable required review for a particular scan, all lobes in which discrepancy was seen between original readers were reviewed by the consensus reader.

Consensus reading was performed by a third independent chest radiologist (HZ). Reviews were performed at the end of the study, and the consensus reader was provided with both the HRCT images and anonymised original reads from each of the primary radiologists. When reviewing discrepant variables/lobes, the reviewer was able to either choose one of the original scores or generate their own response. For pragmatic reasons, no consensus review was performed for pleural pathology (final readings were taken from one reader only) and lymphadenopathy for which non-contrast imaging is known to have limited sensitivity and which was shown to have very low inter-reader agreement suggesting poor validity of data.

Table E4: Methods used to select scans and variables for consensus review, including the difference in scores used as a cut-off for consensus reading, the number of scans reviewed and the approach underlying this decision given.

Variable group	Variable<br>(Range of possible scores, whole lung level)	Inter-reader consistency for whole-lung variable<br>ICC (95% CI) OR Kappa (SE)	Threshold difference, at which consensus review required	Number of scans for review of ≥1 lobe	Number of lobes for which clean reads available (RUL/RML/RLL – LUL/Lingula/LLL)<br><br>n (%) of scans with all lobes having clean reads††
Parenchymal variables	Atelectasis score (0-600)	ICC:<br>0.81 (0.77-0.84)	≥60 points	20	R lobes: 308 / 330 / 334<br>L lobes: 308 / 321 / 332<br>Clean reads all lobes: 177/385 (46.0%)
	Parenchymal banding score (0-600)	ICC:<br>0.43 (0.35 -0.51)	≥75 points	22	R: 221/204/232<br>L: 231 / 244/ 238

					Clean reads all lobes: 86/385 (22.3%)
	Consolidation score (0-600)	ICC:<br>0.43 (0.34 – 0.51)	≥40 points	18	R 311 / 346 / 338<br>L: 301 / 338 / 342<br>Clean reads all lobes: 194/385 (50.4%)
	Ground glass opacification score (0-600)	ICC:<br>0.49 (0.41 – 0.57)	≥60 points	22	R: 353 / 367 / 366<br>L: 362/366 / 360<br>Clean reads all lobes: 292/385 (75.8%)
	Mosaicism score (0-600)	ICC:<br>0.55 (0.48-0.62)	≥160 points	22	R: 236 / 242/232<br>L: 234 / 259 / 226<br>Clean reads all lobes:105/385 (27.3%)
	Emphysema score (0-600)	ICC:<br>0.50 (0.42 – 0.57)	≥25 points	18	R: 335 / 372/ 364<br>L: 339 / 375 / 363<br>Clean reads all lobes:291/385 (75.6%)
	Emphysematoid destruction score (0-600) †	ICC:<br>0.27 (0.18 – 0.36)	≥15 points	18	R: 379 / 380 / 378<br>L: 374 / 384 / 371<br>Clean reads all lobes: 346/385 (89.9%)
	Cavities score (0-600)	ICC:<br>0.81 (0.77-0.84)	≥25 points	23	R: 344 / 378 / 370<br>L: 349 / 380 / 375<br>Clean reads all lobes: 301/385 (78.2%)
	Normal lung score (0-600)	ICC:<br>0.80 (0.76 – 0.83)	≥170 points	21	R:183 / 161 / 172<br>L: 196 / 208 /186<br>Clean reads all lobes: 73/385(19.0%)
Bronchiectasis	Whole lung bronchiectasis extent score (0-16)	Weighted kappa: 0.72 (0.05)	≥6 points	20	R: 296 / 340/ 324<br>L: 297 / 337 / 321<br>Clean reads all lobes: 195/385 (50.7%)
	Whole lung bronchiectasis severity score (0-18)	Weighted kappa: 0.66 (0.05)	≥6 points	25	R: 281 / 315 / 299<br>L: 266 / 307 / 292<br>Clean reads all lobes: 133/385 (34.6%)
	Whole lung bronchiectasis pattern score (0-18)*	Weighted kappa: 0.67 (0.05)	≥6 points	20	R: 41/78 (52.6%); 7/15 (46.7%); 16/35 (45.7%)<br>L: 42/80 (52.5%); 29/39 (74.4%); 25/39 (64.1%)
	Whole lung presence / absence of bronchiectasis ‡	N/a	All with discrepancy	79	N/a
	Number of lobes affected (0-6) ‡	N/a	≥3 lobes	30	N/a
Cavities / cystic airspaces	Whole lung cavity /cystic airspace extent score (0-18)	Weighted kappa: 0.65 (0.04)	≥4 points	23	R: 359 / 383 / 372<br>L: 362 / 380 / 371<br>Clean reads all lobes: 327/385 (84.9%)
	Whole lung presence / absence of cavities or cystic airspaces ‡	N/a	All with discrepancy	58	N/a
	Whole lung presence / absence of mycetoma	Kappa:<br>0.49 (0.05)	All with discrepancy	10	R: 98/100 (98.0%); 16/16 (100%); 32/32 (100%)<br>L: 103/105 (98.1%); 25/26 (96.2%); 44/44 (100%)
	Maximum cavity size (size in mm)	N/a	≥22mm	20	N/a
	Parenchymal change allocated to cavities/cystic	N/a	≥10% parenchyma in lobe	7	N/a

	airspaces, but no cavities seen, in at least 1 lobe §				
	Cavity seen, but no % parenchyma allocated, in at least 1 lobe §	N/a	≥10mm cavity seen in lobe	26	N/a
Nodules	Whole lung nodule extent score (0-18)	Weighted kappa: 0.65 (0.05)	≥5	22	R: 267 / 320 / 304<br>L: 274 / 315/ 291<br>Clean reads all lobes: 115/385 (29.9%)
Other airway variables	Whole lung tree in bud severity score (0-18)	Weighted kappa: 0.45 (0.04)	≥9	20	R: 217 / 247 / 226<br>L: 219 / 248 / 225<br>Clean reads all lobes: 67/385 (17.4%)
	Whole lung airway plugging severity score (0-18)	Weighted kappa: 0.51 (0.05)	≥5	18	R: 284 / 328 / 302<br>L: 280 / 313 / 313<br>Clean reads all lobes: 153/385 (39.7%)
	Whole lung bronchial wall thickening severity score (0-18)	Weighted kappa: 0.42 (0.05)	≥8	22	R: 193 / 248 / 228<br>L: 198 / 226 / 205<br>Clean reads all lobes: 73/385 (19.0%)
Whole lung variables	Presence /absence of pleural pathology ‖	Kappa: 0.60 (0.05)	N/a	0	368/385 (95.6%)
	Presence /absence of lymphadenopathy‖	Kappa: 0.17 (0.05)	N/a	0	339/385 (88.1%)
All variables	Total number of CT scans requiring consensus review of ≥1 lobe		239 scans		

† Term derived from TB imaging literature, and not Fleischner defined.

*Bronchiectasis pattern is an ordinal variable, but lobar scores treated as nominal and summed / compared to identify scans for consensus review only.

‡ Derived measures of pathology, for which no measures of inter-reader agreement were calculated.

§ Discrepancies within scoring tool, with differences in the reporting of the extent of cavities / cystic airspaces between sections.

‖ No consensus review performed: final scores derived from a single reader only (pleural pathology), or data not used given very low levels of inter-reader agreement (lymphadenopathy).

††Clean reads: lobes with either identical scores from original readers, or score determined by consensus review

Final dataset development

Original and consensus reads were combined to form a single 'final' dataset. Where there was agreement between original readers for a given variable in a given lobe, this score was used as the final data point. Where original readers had disagreed and a consensus review had been performed, the consensus score was used as the final data point. Where original readers had disagreed, but no consensus read was available, the approach taken varied: scores from the original readers were averaged for continuous variables (eg. parenchymal scores), pathology was considered present if either or both original readers felt it to be so for binary scores (eg. mycetoma, pleural pathology),

Meghji J, *et al. Thorax* 2020; 75:1–10. doi: 10.1136/thoraxjnl-2019-213808

and random selection of original reads was used for nominal variables which could not be combined (eg. bronchiectasis pattern).

Lobar scores were summed to generate whole-lung level scores for data analysis. This pragmatic approach assumes that the impact of pathology in all lobes is equal, and that each lobe contributes the same volume of parenchyma / pathology to the overall lung, regardless of variation in their true size. Several new variables were derived including: lobar presence / absence scores for airway pathologies (E.g. moderate to severe bronchiectasis was considered present in a lobe if the final score was ≥2), a composite variable for 'destroyed lobes' (≥90% of parenchyma was occupied by atelectasis, parenchymal banding or destroyed by cavities/cystic airspaces), and a % score for the total amount of abnormal parenchyma seen across the lung both with and without mosaicism.

The data collection team and participants remained blind to imaging results until after the final study visit, at which point written imaging reports were provided to each participant, and findings explained to each individual by a respiratory physician.

Appendix 4: Sample size calculation

An *a priori* definition of severe PTLD was established prior to data analysis, based on parameters known to predict adverse patient outcomes in other chronic lung diseases (bronchiectasis, COPD, and ILD) and consensus discussion between a panel of respiratory and TB researchers and clinicians[1](Table 5). The prevalence of severe PTLD was estimated at between 10-50%. A sample size of 400 was sufficient to determine the prevalence of severe PTLD within this range with +/-5% precision and 95% confidence.

Table E5: Composite *a priori* definition of PTLD

Criteria	PTLD present
Abnormal spirometry<br>OR	Airway obstruction with FEV1/FVC ratio<LLN & FEV1<LLN<br>OR<br>Low FVC with FEV1/FVC ratio≥0.7 & FVC<LLN
Abnormal CT Imaging	Moderate-severe bronchiectasis in ≥3 lobes<br>OR<br>Parenchymal abnormality of ≥1/3 of the lung tissue, excluding mosaicism

LLN: Lower limit of normal, as classified using GLI-2012 reference ranges.

The rationale for the parameters and cut-offs included in the *a priori* definition are given below. The studies on which these findings are based include data from non-TB populations in high-resource settings, but was felt plausible that similar outcomes may be seen in in the post-TB population in Malawi also.

Spirometry criteria:

Use of the LLN to define abnormal spirometry is widely accepted practice.(3) Reduced FEV_1 volumes predict mortality both amongst those with COPD, and the general population, with causes including respiratory failure and cardiovascular disease.(8) Recent data suggest that the FVC may be an

---

[1] JM, SBS, PM, Professor Jane Carter (Respiratory physician and Associate Professor of Medicine at Brown University (USA), past-president of The International Union Against Tuberculosis and Lung Disease), Dr Jeremiah Chakaya (Respiratory physician, lead of Kenyan National TB Control Programme, past-president of The International Union Against Tuberculosis and Lung Disease)

Meghji J, *et al. Thorax* 2020; 75:1–10. doi: 10.1136/thoraxjnl-2019-213808

additional, or even more important, driver of mortality even in the absence of persistent symptoms or an underlying diagnosis of lung pathology.(9, 10) Both airway obstruction with reduced FEV_1, and small lungs with reduced FVC were therefore included.

Imaging criteria:

Bronchiectasis and parenchymal damage are common features of PTLD.(5) Bronchiectasis affecting ≥3 lobes on CT imaging has been associated with hospital admissions and all-cause mortality in previous bronchiectasis cohort studies.(11, 12) Studies developing prognostic tools for patients with emphysema and scleroderma related ILD suggest that a cut-off of 20-30% abnormal parenchyma can help to differentiate between those at low and moderate risk of mortality.(13, 14) Because the relative importance of each pattern of parenchymal pathology in PTLD is not known, a cumulative variable including all patterns of parenchymal pathology was used in the definition given here. The extent of mosaicism, which reflects gas trapping rather than parenchymal damage, was not included.

Meghji J, *et al. Thorax* 2020; 75:1–10. doi: 10.1136/thoraxjnl-2019-213808

Appendix 5: Comparison of baseline study spirometry data with community data

Table E6: Comparison of the age-stratified prevalence estimates of moderate-severe airway obstruction and spirometric restriction within this study cohort, with survey weighted prevalence estimates from the 2013-2014 BOLD study in urban Blantyre.(15) All data standardised using NHANES III Caucasian reference ranges.

Age group (n at baseline/ 1-year)	Mod-severe obstruction FEV/FVC<0.7 and FEV1<80% predicted			Low FVC FEV1/FVC>=0.7 and FVC<80% predicted		
	Post-TB cohort TB Rx completion % (SE)	Post-TB cohort 1-year f'up % (SE)	BOLD data set % (SE)	Post-TB cohort TB Rx completion % (SE)	Post-TB cohort 1-year f'up % (SE)	BOLD data set % (SE)
15-19yrs (n=17/13)	11.8% (7.8%)	15.4% (10.0%)	-	82.4% (9.2%)	61.5% (13.5%)	-
20-29yrs (n=83/77)	4.8% (2.4%)	6.5% (2.8%)	2.4% (1.0%)	71.1% (5.0%)	64.9% (5.4%)	32.7% (4.0%)
30-39yrs (n=160/147)	7.5% (2.1%)	8.8% (2.3%)	2.9% (1.5%)	64.4% (3.8%)	55.8% (4.1%)	42.0% (3.8%)
40-49yrs (n=79/75)	11.4% (3.6%)	16.0% (4.2%)	2.3% (0.9%)	62.0% (5.5%)	41.3% (5.7%)	28.3% (4.4%)
50-59yrs (n=17/15)	11.8% (7.8%)	13.3% (8.8%)	9.8% (2.8%)	17.6% (9.2%)	6.7% (6.4%)	39.4% (6.6%)
60+yrs (n=9/9)	11.1% (10.5%)	33.3% (15.7%)	12.0% (4.4%)	33.3% (15.7%)	33.3% (15.7%)	13.8% (6.4%)

Meghji J, *et al. Thorax* 2020; 75:1–10. doi: 10.1136/thoraxjnl-2019-213808

Appendix 6: Clinical and respiratory parameters, stratified by HIV-status

Table E7: Clinical and respiratory parameters measured at TB treatment completion, 6-month and 12-month study visits, stratified by HIV-status [§] (n=403)

Parameter	TB treatment completion			6-month visit			12-month visit		
	HIV-negative (n=159)	HIV-positive (n=244)	p-value	HIV-negative (n=143)	HIV-positive (n=231)	p-value	HIV-negative (n=142)	HIV-positive (n=225)	p-value
Symptom prevalence (n, %) [†]									
Breathlessness									
- Never/only with chest infections	83 (52.2%)	143 (58.6%)	0.069	96 (67.1%)	186 (80.5%)	0.014*	101 (71.1%)	181 (80.4%)	0.086
- Few days per month	65 (40.9%)	95 (38.9%)		40 (28.0%)	38 (16.5%)		35 (24.7%)	40 (17.8%)	
- ≥Several days per week	11 (6.9%)	6 (2.5%)		7 (4.9%)	7 (3.0%)		6 (4.2%)	4 (1.8%)	
Cough									
- Never/only with chest infections	86 (54.1%)	172 (70.5%)	0.002*	106 (74.1%)	177 (76.6%)	0.860	113 (79.6%)	194 (86.2%)	0.233
- Few days per month	66 (41.5%)	68 (27.9%)		31 (21.7%)	45 (19.5%)		26 (18.3%)	27 (12.0%)	
- ≥Several days per week	7 (4.4%)	4 (1.6%)		6 (4.2%)	9 (3.9%)		3 (2.1%)	4 (1.8%)	
Sputum production									
- Never/only with chest infections	109 (68.6%)	190 (77.9%)	0.077	113 (79.0%)	186 (80.5%)	0.940	118 (83.1%)	200 (88.9%)	0.240
- Few days per month	45 (28.3%)	51 (20.9%)		28 (19.6%)	42 (18.2%)		23 (16.2%)	23 (10.2%)	
- ≥Several days per week	5 (3.1%)	3 (1.2%)		2 (1.4%)	3 (1.3%)		1 (0.7%)	2 (0.9%)	
Wheeze									0.235
- Never/only with chest infections	147 (92.5%)	223 (91.4%)	0.823	130 (90.9%)	215 (93.1%)	0.188	134 (94.4%)	218 (96.9%)	
- Few days per month	11 (6.9%)	18 (7.4%)		11 (7.8%)	16 (6.9%)		8 (5.6%)	7 (3.1%)	
- ≥Several days per week	1 (0.6%)	3 (1.2%0		2 (1.4%)	0 (0 %)		0 (0%)	0 (0%)	
Any respiratory symptom, ≥monthly	103 (64.8%)	142 (58.2%)	0.186	62 (43.4%)	75 (32.5%)	0.034*	55 (38.7%)	57 (25.3%)	0.007*
Symptom impact (n, %)									
Self-reported impact of chest on activities									
- Does not stop any activities	77 (48.4%)	122 (50.0%)	0.837	108 (75.5%)	181 (78.4%)	0.578	108 (76.1%)	187 (82.1%)	0.241
- Prevents 1-2 activities	67 (42.1%)	97 (39.8%)		27 (28.9%)	41 (17.8%)		28 (19.7%)	28 (12.4%)	
- Prevents most / all activities	15 (9.4%)	25 (0.10%)		8 (5.6%)	9 (3.9%)		6 (4.2%)	10 (4.4%)	
Self-reported impact of chest on work									
- Does not affect work	95 (59.8%)	146 (59.8%)	0.964	115 (80.4%))	192 (83.1%)	0.797	122 (85.9%)	200 (88.9%)	0.022
- Interferes with / made me change work	58 (36.5%)	90 (36.9%)		24 (16.8%)	33 (14.3%0		20 (14.1%)	18 (8.0%)	
- Made me stop work	6 (3.8%)	8 (3.3%)		4 (2.8%)	6 (2.6%)		0 (0%)	7 (3.1%)	
Breathless at rest / during personal care	0 (0%)	2 (0.8%)	0.253	1 (0.7%)	1 (0.4%)	0.731	1 (0.7%)	1 (0.4%)	0.742
Walks slower than peers / stops for rest at own pace	45 (28.5%)	62 (25.5%)	0.512	26 (18.2%)	31 (13.4%)	0.213	28 (19.7%)	35 (15.6%)	0.303
Breathless on hills	79 (50.0%)	96 (39.5%)	0.038*	42 (29.4%)	39 (16.9%)	0.004*	40 (28.2%)	42 (18.9%)	0.033*
Quality of life									
Self-reported general health (n, %)									
- Poor/fair	45 (28.3%)	70 (28.7%)	0.933	23 (16.1%)	30 (13.0%)	0.404	9 (6.3%)	13 (5.8%)	0.826
- Good/excellent	114 (71.7%)	174 (71.3%)		120 (83.9%)	201 (87.0%)		133 (93.7%)	212 (94.2%)	
SGRQ Total score (median, IQR)	10.3 (1.8 – 24.7)	8.2 (0.9 – 22.4)	0.1135	1.1 (0.4 – 16.4)	0.4 (0.0 – 7.3)	0.1043	0.4 (0 – 11.6)	0.4 (0 – 4.6)	0.1300
SGRQ Symptom score (median, IQR)	13.7 (2.7 – 28.8)	10.3 (2.7 – 21.8)	0.0185*	2.7 (0 – 21.9)	2.7 (0 – 10.7)	0.0991	2.7 (0 – 21.4)	2.7 (0 – 10.3)	0.1204

SGRQ Activity score (median, IQR)	12.2 (0 – 41.4)	11.2 (0 – 35.2)	0.1212	0 (0 – 24.1)	0 (0 – 11.2)	0.0279*	0 (0 - 18.2)	0 (0 – 0)	0.0994
SGRQ Impact score (median, IQR)	7.3 (0 – 15.5)	5.5 (0 – 15.0)	0.2777	0 (0 – 7.3)	0 (0 – 5.6)	0.1411	0 (0 – 5.6)	0 (0 – 1.6)	0.0214*
Clinical observations									
BMI (kg/m^2) (median, IQR)	20.3 (18.8 – 21.7)	20.7 (19.0 – 22.6)	0.0471*	20.5 (19.0 – 22.1)	21.2 (19.6 – 23.3)	0.0055*	20.7 (19.2 – 22.5)	21.5 (19.6 – 23.8)	0.0060*
Oxygen saturations (%) (median, IQR)	98 (97 – 99)	98 (98-99)	0.0042*	98 (97-98)	98 (97 – 99)	0.0327*	98 (97-98)	98 (97 – 98)	0.2691
Hypoxaemia (sats <92%) (n, %)	4 (2.5%)	2 (0.8%0	0.169	4 (2.8%)	2 (0.9%)	0.149	3 (2.1%)	1 (0.4%)	0.134
Respiratory rate (breaths/minute) (median, IQR)	19 (17 – 20)	18 (17 – 20)	0.9749	19 (18 – 21)	19 (18 – 21)	0.3201	20 (19 – 22)	20 (19 – 22)	0.8404
Heart rate (beats/minute) (median, IQR)	73 (64 – 86)	82 (72 – 91)	0.0000*	73 (65 – 86)	77 (69 – 86)	0.0117*	73 (6 3- 84)	78 (70 – 87)	0.0004*
Pedal oedema (n, %)	1 (0.6%)	6 (2.5)	0.169	1 (0.7%)	2 (0.9%)	0.861	0 (0%)	3 (1.3%)	0.167
Palatal Kaposi Sarcoma (n=368) (n, %)	2 (1.4%)	6 (2.7%)	0.418	1 (0.7%)	9 (3.9%)	0.063	0 (0.0%)	1 (0.4%)	0.426
Blood tests									
Haemoglobin (g/dL) (median, IQR)	14.6 (13.3 – 15.6)	13.1 (11.7 – 14.5)	0.0000*						
Positive aspergillus IgG ELISA	1 (0.6%)	2 (0.8%)	0.828				0 (0%)	2 (0.8%)	0.252
6-minute walk test (n=395 / 355)									
Distance (m) (mean, sd)	576 (524 – 627)	570 (508 – 617)	0.3360				624 (576 – 663)	606 (564 – 654)	0.1149
Spirometry (n=365 / 341 / 336) ‡									
FEV_1 z-score (mean, sd)	-1.27 (1.33)	-0.94 (1.19)	0.0146*	-1.17 (1.33)	-0.74 (1.17)	0.0019*	-1.15 (1.28)	-0.70 (1.10)	0.0007*
FVC z-score (mean, sd)	-1.08 (1.29)	-0.80 (1.18)	0.0374*	-0.92 (1.29)	-0.50 (1.10)	0.0013*	-0.86 (1.16)	-0.44 (1.02)	0.0006*
FEV_1/FVC ratio z-score (mean, sd)	-0.47 (1.40)	-0.32 (1.15)	0.2492	-0.54 (1.49)	-0.49 (1.14)	0.7310	-0.62 (1.45)	-0.49 (1.19)	0.3728
Pattern of spirometry<br>- Obstruction (FEV1/FVC ratio <LLN)<br>- Low FVC (FEV1/FVCratio≥LLN & FVC<LLN)<br>- Normal (FEV1/FVC ratio ≥LLN & FVC≥LLN)	 27 (19.0%)<br>36 (25.4%)<br>79 (55.6%)	 25 (11.3%)<br>37 (16.7%)<br>159 (72.0%)	 0.006*	 29 (22.7%)<br>23 (18.0%)<br>76 (59.4%)	 31 (14.6%)<br>22 (10.4%)<br>159 (75.0%)	 0.010*	 28 (21.9%)<br>23 (18.0%)<br>77 (60.2%)	 31 (15.0%)<br>20 (9.7%)<br>156 (75.4%)	 0.011*
CXR findings (n=403 / 361)									
% Abnormal parenchyma (median, IQR)	4.6 (0.8 – 13.3)	1.7 (0 – 7.9)	0.0000*				4.0 (0.8 – 10.8)	1.0 (0.0 – 5.0)	0.0000*
Ring and tramline severity score (0-18) (median, IQR)	2.0 (0.0 – 3.5)	0.5 (0.0 – 2.5)	0.0005*				1.5 (0.0 – 3.0)	0.5 (0.0 – 2.0)	0.0065*

*Statistically significant difference between HIV-positive and negative groups, at p<0.05 level.

†Symptom questions derived from SGRQ: Over the past 3-months I have (had shortness of breath / coughed / brought up sputum / had attacks of wheezing): not at all / only with chest infections / a few days a month / several days a week / most days a week; If you have tried to work in the past 3-months: my chest trouble does not affect my work / my chest trouble interferes with my work or made me change my work / my chest trouble made me stop work; Which of these statements best describes how your chest affects you: It does not stop me doing anything I would like to do / It stops me doing 1-2 things I would like to do / it stops me doing most of the things I would like to do / It stops me doing everything I would like to do.

‡ BOLD standard data available for n=365/405 at baseline, n=341/376 at 6-months, and n=336/368 at 12-month study visits. Data age / sex / height standardised using GLI 2012 African American reference ranges to generate z-scores.

§ Data compared between HIV groups using Chi^2 for categorical, and Student's t-test / Wilcoxon rank sum for continuous variables.

Appendix 7: CT imaging data, stratified by HIV-status

Table E8: Final CT imaging data, generated from original and consensus reads, stratified by HIV status (n=383 individuals)

Pathology	All scans (n=385) Median (IQR) [Full range], or N (%)	HIV-negative (n=148) Median (IQR) [Full range] or N (%)	HIV-positive (n=235) Median (IQR) [Full range] or N (%)	p-value
% parenchymal pathology , whole lung level				
Atelectasis and banding	7.5 (2.9 – 14.2) [0.0 – 53.3]	9.2 (5.8 – 17.5) [0.0 – 53.5]	5.8 (2.5 – 11.7) [0.0 – 43.3]	0.0000*
Cavities / cystic air spaces	0.0 (0.0 – 1.7) [0.0 – 46.7]	0.8 (0.0 – 2.7) [0.0 – 46.7]	0.0 (0.0 – 0.8) [0.0 – 27.5]	0.0001*
Mosaicism	5.4 (0.8 – 14.2) [0.0 – 54.2]	6.7 (2.1 – 15.2) [0.0 – 42.1]	4.6 (0.4 –12.5) [0.0 – 54.2]	0.0240*
Emphysema	0.0 (0.0 – 0.8) [0.0 – 71.7]	0.0 (0.0 – 0.4) [0.0 – 64.2]	0.0 (0.0 – 0.8) [0.0 – 71.7]	0.0161*
Ground glass	0.0 (0.0 – 0.8) [0.0 – 45.0]	0.0 (0.0 – 0.8) [0.0 – 27.5]	0.0 (0.0 – 0.8) [0.0 – 45.0]	0.2759
Consolidation	0.8 (0.0 – 2.1) [0.0 – 18.3]	0.8 (0.0 – 2.1) [0.0 – 18.3]	0.8 (0.0 – 2.1) [0.0 – 12.5]	0.1279
Emphysematous destruction	0.0 (0.0 – 0.4) [0.0 – 27.5]	0.0 (0.0 – 0.8) [0.0 – 20.0]	0.0 (0.0 – 0.4) [0.0 – 27.5]	0.1186
Total abnormal parenchyma, any pattern	22.9 (9.2 – 39.2) [0.0 – 100.0]	30.0 (13.5 –41.0) [0.0 – 100.0]	18.3 (6.7– 37.9) [0.0 – 95.8]	0.0003*
Total abnormal parenchyma, excluding mosaicism†	12.1 (5.0 – 25.0) [0.0 – 100.0]	15.6 (7.9 – 30.0) [0 – 100.0]	10.0 (3.8 – 21.7) [0 – 90.0]	0.0003*
Number of 'destroyed' lobes ‡<br>- 0<br>- 1-2<br>- 3	 349 (90.7%)<br>33 (8.6%)<br>3 (0.8%)	 126 (85.1%)<br>19 (12.8%)<br>3 (2.0%)	 222 (94.5%)<br>13 (5.5%)<br>0 (0%)	 0.009*
Airway scores, whole lung level				
Bronchiectasis extent score (0-16)	2.5 (0.5 – 4.5) [0.0 – 15.5]	3.0 (2.0 – 5.5) [0 – 15.5]	1.5 (0.0 – 4.) [0.0 – 13.5]	0.0000*
Bronchiectasis severity score (0-18)	2.5 (0.5 – 5.0) [0.0 – 15.5]	3.5 (1.5 – 6.0) [0.0 – 15.5]	2.0 (0.0 – 4.0) [0.0 – 14.5]	0.0000*
Bronchial wall thickening severity score (0-18) (n=327) §	3.0 (1.5 – 5.5) [0.0 – 14.5]	3.0 (1.5 – 5.5) [0.0 – 13.5]	3.0 (1.0 – 6.0) [0.0 – 14.5]	0.3883
Tree in bud severity score (0-18)	3.5 (1.5 – 6.0) [0.0 – 17.0]	4.5 (2.5 – 6.5) [0.0 – 14.5]	3.0 (1.0 – 6.0) [0.0 – 17.0]	0.0006*
Airway plugging severity score (0-18)	1.0 (0.0 – 2.0) [0.0 – 12.0]	1.0 (0.5 – 2.5) [0.0 – 9.0]	1.0 (0.0 – 2.0) [0.0 – 12.0]	0.0196*
Number of lobes with moderate – severe bronchiectasis **<br>- 0<br>- 1-2<br>- ≥3	 215 (55.8%0<br>141 (36.6%)<br>29 (7.5%)	 65 (43.9%)<br>71 (48.0%)<br>12 (8.1%)	 149 (63.4%)<br>69 (29.4%)<br>17 (7.2%)	0.001*
Any moderate – severe cystic bronchiectasis ††	49 (12.7%)	28 (18.9%)	20 (8.5%)	0.0030*
Other variables, whole lung level ‡‡				
Mycetoma present	5 (1.3%) §§	4 (2.7%)	1 (0.4%)	0.0560
Nodules present	228 (59.2%)	88 (59.5%)	139 (59.2%)	0.1540
Pleural pathology (effusions or thickening) present	31 (8.1%)	12 (8.1%)	19 (8.1%)	0.9940

*Statistically significant difference between HIV-positive and negative groups, at p<0.05 level.

†Mosaicism excluded as represents areas with gas trapping or impaired perfusion rather than primary parenchymal pathology, and can be observed in 'normal' health adults.

‡ Destroyed lobe: lobe with ≥90% of parenchyma is occupied by banding, atelectasis, or cavities / cystic airspaces.

Meghji J, *et al. Thorax* 2020; 75:1–10. doi: 10.1136/thoraxjnl-2019-213808

[§] Data missing where extensive parenchymal pathology in ≥1 lobe prevented evaluation of bronchial wall thickness – those with missing data had more abnormal parenchyma (median 46.3% vs. 18.8% in th, p<0.001) and a lower prevalence of HIV-infection (35.9% vs. 54.0%, p=0.004) compared to those with data for this variable.

** Present if average lobar severity score between two readers, or consensus score, was ≥2, so on average bronchial lumen considered to be 2-3 times adjacent vessel diameter in these lobes.

[††] Present if moderate to severe bronchiectasis seen in at least 1 lobe, and pattern here deemed to be cystic based on initial agreement between readers or consensus review of scans, or random selection of initial reader reports where disagreement seen and no consensus review available.

[‡‡]Mycetoma / nodules present if confirmed by both original readers or the consensus scorer. Pleural pathology present if reported by either original reader.

[§§] All patients with mycetoma had negative aspergillus IgG at TB treatment completion.

Appendix 8: Relationship between symptoms and spirometry and imaging findings, at TB treatment completion

Table E9: Relationship between spirometry and CT parameters, and symptoms and quality of life at TB treatment completion with p-values for association

Symptom / QoL Parameter	Prevalence (n, %)	Spirometry parameters (n=365)			CT imaging parameters (median, IQR) (n=385)		
		FEV_1 z-score (mean, SD)*	FVC z-score (mean, SD)*	FEV_1/FVC ratio z-score (mean, SD)*	Bronchiectasis severity score (0-18) (median, IQR)†	% abnormal parenchyma (median, IQR) †	Presence of ≥1 destroyed lobe (n, %, 95% CI)‡
Breathlessness							
- Never/only with chest infections	227 (56.0%)	-0.86 (1.18)	-0.72(1.17)	-0.31 (1.09)	2.5 (1.0 – 4.5)	21.9 (9.2 – 35.4)	16 (7.3%) (4.3 -11.6%)
- ≥ Few days per month	178 (44.0%)	-1.32 (1.31)	-1.14 (1.27)	-0.45 (1.44)	2.5 (0.5 – 5.5)	27.1 (9.6 – 42.9)	20 (12.0%) (7.5 -17.9%)
		p<0.001	p=0.001	p=0.287	p=0.440	p=0.070	p=0.051
Cough							
- Never/only with chest infections	259 (64.0%)	-0.94 (1.22)	-0.84 (1.18)	-0.24 (1.23)	2.0 (0.5 – 4.5)	18.8 (7.1 – 35.0)	13 (5.3%) (2.9-8.9%)
- ≥ Few days per month	146 (36.0%)	-1.27 (1.30)	-1.02 (1.31)	-0.61 (0.11)	3.0 (1.0 – 5.5)	31.3 (12.9 – 48.3)	23 (16.4%) (10.7-23.6%)
		p=0.015	p=0.176	p=0.006	p=0.014	P<0.001	p<0.001
Sputum production							
- Never/only with chest infections	300 (74.1%)	-1.01 (1.23)	-0.89 (1.19)	-0.26 (1.26)	2.0 (0.5 – 4.50)	20.8 (8.3 – 38.8)	24 (8.4%) (5.5-12.3%)
- ≥ Few days per month	105 (25.9%)	-1.22 (1.33)	-0.94 (1.34)	-0.68 (0.12)	2.5 (1.0 – 5.25)	28.1 (13.5 – 42.5)	12 (12.0%) (6.4-20.0%)
		p=0.156	p=0.728	p=0.005	p=0.089	p=0.009	p=0.558
Wheeze							
- Never/only with chest infections	372 (91.8%)	-1.05 (1.26)	-0.92 (1.20)	-0.33 (1.22)	2.5 (0.5 – 5.0)	24.2 (9.2 – 39.6)	34 (9.6%) (6.7-13.1%)
- ≥ Few days per month	33 (8.2%)	-1.23 (1.33)	-0.72 (0.30)	-0.88 (0.29)	2.0 (0.5 – 3.0)	20.4 (9.6 – 38.8)	2 (6.7%) (0.8-22.1%)
		p=0.451	p=0.4071	p=0.0260	p=0.220	p=0.850	p=0.599
Any respiratory symptom							
- Never/only with chest infections	159 (39.3%)	-0.79 (1.19)	-0.68 (1.17)	-0.25 (1.12)	2.0 (0.5 – 4.0)	18.3 (7.1 – 34.6)	7 (4.6%) (1.9-9.3%)
- ≥ Few days per month	246 (60.7%)	-1.23 (1.28)	-1.05 (1.25)	-0.45 (1.34)	2.5 (0.5 – 5.0)	27.3 (10.0-42.9)	29 (12.4%) (8.5-17.3%)
		p=0.001	p=0.013	p=0.130	p=0.349	p=0.002	p=0.011

*Student's t-test; †Wilcoxen rank sum test; ‡Chi-square test

Appendix 9: Multi-level linear regression models for change in spirometry over time

Table E10: Multi-level linear regression, to investigate parameters predicting spirometry values in the first year after TB treatment completion† (n=347).‡

Variable measured at TB treatment completion	Univariate (ml, 95% CI)	Multivariate, partial model (ml, 95% CI)	Multivariate, full model (ml, 95% CI)
Absolute FEV_1 (ml) over follow-up period			
Time from TB treatment end §			
6-months	66.70 (47.39 – 86.01)*	62.17 (41.78 – 82.56)*	65.30 (45.00 – 85.61)*
12-months	72.73 (48.26 – 97.19)*	65.57 (39.47 – 91.68)*	70.56 (44.58 – 96.54)*
HIV positive status	197.57 (83.03 – 312.11)*	193.75 (79.43 – 308.08)*	98.61 (-2.01 – 199.22)
Microbiologically proven TB	-61.81 (-194.79 – 71.17)	-9.12 (-140.23 – 121.99)	30.82 (-84.35 – 145.98)
BMI (kg/m2)	18.32 (9.26 – 27.38)*	7.39 (-1.96 – 16.74)	2.20 (-7.01 – 11.40)
Pack-year smoking history	-7.90 (-20.00 – 4.20)	-4.90 (-16.79 – 7.00)	-0.75 (-11.15 – 9.65)
Maximum education ≤ 1ry school	-108.59 (-225.94 – 8.76)	-108.26 (-224.69 – 8.18)	-37.49 (-139.97 – 64.99)
Respiratory symptoms ≥monthly	-198.98 (-310.07 - -87.90)*		-111.26 (-208.10 - -14.43)*
Bronchiectasis severity score (0-18) – 3-point increments ‖	-221.04 (-270.18 - -171.91)		-95.56 (-155.64 - -35.47)*
Abnormal parenchyma (%) – 10% increments **	-152.87 (-180.38 - -125.36)		-106.40 (-141.38 - -71.4)*
Variance components (% of model variance): change over time		*1.85%*	*2.53%*
Variance components (% of model variance): baseline FEV1		*94.22%*	*92.05%*
Absolute FVC (ml) over follow-up period			
Time from TB treatment end §			
- 6-months	124.49 (100.70 – 148.30)*	111.77 (87.04 – 136.50)*	115.38 (90.72 – 140.05)*
- 12-months	145.63 (117.66 – 173.59)*	125.21 (95.26 – 155.16)*	131.28 (101.41 – 161.15)*
HIV positive status	197.30 (75.42 – 319.18)*	184.22 (64.02 – 304.43)*	92.94 (-18.17 – 204.04)
Microbiologically proven TB	-18.24 (-159.57 – 123.09)	30.45 (-107.28 – 168.17)	65.99 (-61.11 – 193.09)
BMI (kg/m2)	40.58 (29.97 – 51.19)*	21.34 (10.73 – 31.94)*	15.35 (4.75 – 25.95)*
Pack-year smoking history	-6.04 (-18.88 – 6.81)	-4.38 (-16.86 – 8.10)	-1.87 (-13.34 – 9.59)
Maximum education ≤ 1ry school	-1.34 (-126.72 – 124.04)	-2.97 (-125.46 – 199.51)	63.45 (-49.78 – 176.68)
Respiratory symptoms ≥monthly	-200.30 (-318.52 - -82.08)*		-123.61 (-230.53 - -16.69)*
Bronchiectasis severity score (0-18) – 3-point increments ‖	-217.87 (-271.12 - -164.62)*		-133.62 (-200.01 - -67.23)*
Abnormal parenchyma (%) – 10% increments **	-131.74 (-162.76 - -100.73)*		-67.03 (-105.67 - -28.39)*
Variance components (% of model variance): change over time		*1.60%*	*2.01%*
Variance components (% of model variance): baseline FVC		*93.18%*	*91.26%*

* OR statistically significant at p<0.05 level.

† Model construction based on apriori selection of risk-factors / confounders, and elimination of co-linear variables. Interactions with time evaluated. All univariate & multivariate models coefficients represent the average change in FEV1 or FVC (ml) expected for a 1-unit change in the predictor, holding all other parameters still, and include adjustment for participant age (years), sex, and height (cm).

‡ Participants excluded if absent HIV status at TB treatment completion (n=2), no valid baseline spirometry (n=36), no baseline HRCT imaging (n=20). Includes participants contributing either 6-month (n=13) or 12-month (n=322) follow-up.

§ Negative correlation identified between FEV1 and time (partial model: -0.46 (-0.58 - -0.31) / full model: -0.37 (-0.51 - -0.21)) and FVC and time (partial model: -0.56 (-0.68 - -0.40)/ full model: -0.44 (-0.59 - -0.26)) in all models.

‖ Bronchiectasis severity score (0-18): unweighted sum of 6x lobar bronchiectasis scores ranging from 0 (no bronchiectasis) to 3 (severe dilatation >3 times diameter of adjacent vessel), such that increment of 3 points represents one additional lobe with severe airway dilatation, or combination of 1-3 lobes with less severe disease.

** Total % of parenchymal abnormalities across lung, including: parenchymal banding, atelectasis, consolidation, ground glass change, emphysema, emphysematoid destruction, cavities / cystic airspaces. Excluding mosaicism which represents airway/vascular rather than lung tissue abnormality.

Appendix 10: Predicted change in Spirometry over time

Figure E3: Predicted FEV_1 and FVC volumes at TB treatment completion, and trajectories of change over 1-year, derived from multi-level linear regression models and controlling for patient characteristics, baseline symptoms, and baseline structural lung pathology.

Stratification according to observed change relative to the minimally important clinical difference (MCID) of 100ml.

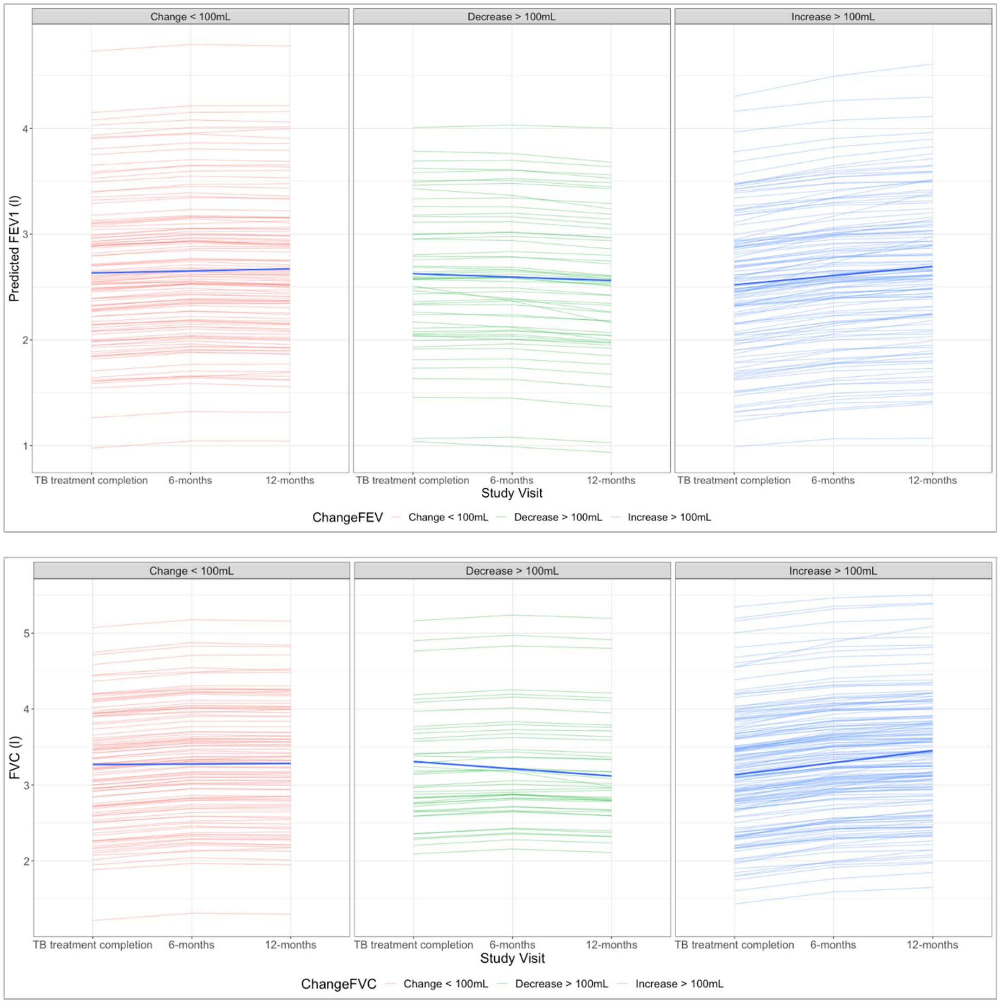

Appendix 11: Factors predicting chronic respiratory symptoms at 1-year, including sensitivity analyses

Table E11: Logistic regression models of baseline parameters associated with the presence of chronic respiratory symptoms† at 1-year (n=325)‡.

Variables measured at TB treatment end	Univariate, OR (95% CI)	Multivariate, controlling for baseline FEV_1§ OR (95% CI)	Multivariate, controlling for baseline FVC § OR (95% CI)
Age (yrs)	0.99 (0.97 – 1.02)	0.98 (0.95 – 1.01)	0.99 (0.96 – 1.01)
Female sex	1.64 (1.00 – 2.70)*	0.63 (0.27 – 1.46)	0.73 (0.31 – 1.71)
Height (cm)	0.96 (0.93 – 0.99)*	0.98 (0.94 – 1.03)	0.97 (0.93 – 1.02)
HIV status			
- Negative	1.0	1.0	1.0
- Positive, CD4≥200	0.46 (0.26 – 0.79)*	0.33 (0.18 – 0.63)*	0.33 (0.18 – 0.62)*
- Positive, CD4<200	0.53 (0.29 – 0.98)*	0.40 (0.19 – 0.84)*	0.38 (0.18 – 0.80)*
Microbiologically proven TB	0.80 (0.46 – 1.40)	0.74 (0.40 – 1.39)	0.74 (0.40 – 1.38)
BMI (kg/m2)	1.01 (0.93 – 1.10)	1.08 (0.98 – 1.19)	1.08 (0.98 – 1.19)
Hb (g/dL)	0.88 (0.78 – 0.995)*	0.82 (0.70 – 0.98)*	0.82 (0.70 – 0.98)*
Pack-year smoking history	0.98 (0.93 – 1.04)	0.98 (0.93 – 1.04)	0.98 (0.93 – 1.04)
Maximum education ≤ 1ry school	1.28 (0.79 – 2.06)	1.18 (0.67 – 2.08)	1.24 (0.71 – 2.17)
Respiratory symptoms ≥monthly	2.74 (1.62 – 4.64)*	2.42 (1.37 – 4.27)*	2.45 (1.39 – 4.32)*
Absolute FEV1 (100 ml increments)	0.93 (0.89 – 0.96)*	0.95 (0.89 – 1.00)	
Absolute FVC (100 ml increments)	0.95 (0.92 – 0.98)*		0.98 (0.93 – 1.03)
Bronchiectasis severity score (3-point increments, 0-6) ‖	1.08 (0.86 – 1.36)	0.88 (0.63– 1.22)	0.89 (0.64 – 1.25)
Abnormal parenchyma (10% increments) ††	1.14 (1.01 – 1.31)*	1.11 (0.91 – 1.35)	1.16 (0.96 – 1.40)

*OR statistically significant at p<0.05 level.

†Cough, breathlessness, sputum production, or wheeze with frequency ≥few days/month.

‡Participants excluded if absent HIV status at TB treatment completion (n=2), no valid baseline spirometry (n=36), no baseline HRCT imaging (n=20), or no symptom data available at 12-month study visit (n=22).

§Co-linearity of FEV_1 and FVC precludes inclusion of both in a single model – separate models constructed to control for baseline level of each.

‖Bronchiectasis severity score (0-18): unweighted sum of 6x lobar bronchiectasis scores ranging from 0 (no bronchiectasis) to 3 (severe dilatation >3 times diameter of adjacent vessel), such that increment of 3 points represents one additional lobe with severe airway dilatation, or combination of 1-3 lobes with less severe disease.

††Total % of parenchymal abnormalities across lung, including: parenchymal banding, atelectasis, consolidation, ground glass change, emphysema, emphysematoid destruction, cavities / cystic airspaces. Excluding mosaicism which represents airway/vascular rather than lung tissue abnormality.

Meghji J, *et al. Thorax* 2020; 75:1–10. doi: 10.1136/thoraxjnl-2019-213808

Table E12: Sensitivity analyses of logistic regression models of baseline parameters associated with the presence of chronic respiratory symptoms † at 1-year (n=347) ‡, with outcomes for those lost to follow-up allocated as positive or negative.

'All symptomatic' models: All participants with missing 1-year symptom data assumed to have ongoing respiratory symptoms (n=22)

'None symptomatic' models: All participants with missing 1-year data assumed to have no ongoing respiratory symptoms (n=22)

Variables measured at TB treatment end	Multivariate model, with FEV_1		Multivariate model, with FVC	
	All symptomatic	None symptomatic	All symptomatic	None symptomatic
Age (yrs)	0.98 (0.95 – 1.01)	0.99 (0.96 – 1.02)	0.98 (0.96 – 1.01)	0.99 (0.97 – 1.02)
Female sex	0.75 (0.34 – 1.65)	0.69 (0.30 – 1.56)	0.87 (0.39 – 1.94)	0.77 (0.34 – 1.76)
Height (cm)	1.00 (0.96 – 1.04)	0.98 (0.93 – 1.02)	0.99 (0.95 – 1.03)	0.97 (0.93 – 1.01)
HIV status				
- Negative	1.0	1.0	1.0	1.0
- Positive, CD4≥200	0.32 (0.18 – 0.59)*	0.39 (0.21 – 0.72)*	0.32 (0.17 – 0.58)*	0.38 (0.21 – 0.71)*
- Positive, CD4<200	0.50 (0.25 – 1.00)	0.41 (0.20 – 0.84)*	0.48 (0.24 – 0.96)*	0.39 (0.19 – 0.80)*
Microbiologically proven TB	0.79 (0.44 – 1.43)	0.75 (0.41 – 1.39)	0.79 (0.44 – 1.42)	0.75 (0.41 – 1.38)
BMI (kg/m2)	1.07 (0.98 – 1.17)	1.08 (0.98 – 1.18)	1.06 (0.97 – 1.17)	1.07 (0.97 – 1.18)
Hb (g/dL)	0.82 (0.70 – 0.96)*	0.88 (0.75 – 1.03)	0.81 (0.70 – 0.95)*	0.88 (0.75 – 1.03)
Pack-year smoking history	0.97 (0.92 – 1.03)	0.99 (0.93 – 1.04)	0.97 (0.92 – 1.03)	0.99 (0.93 – 1.04)
Maximum education ≤ 1ry school	1.07 (0.63 – 1.81)	1.16 (0.67 – 2.00)	1.11 (0.66 – 1.87)	1.21 (0.70 – 2.07)
Respiratory symptoms ≥monthly	2.49 (1.46 – 4.23)*	2.15 (1.23 – 3.75)*	2.54 (1.50 – 4.31)*	2.19 (1.25 – 3.81)*
Absolute FEV1 (100 ml increments)	0.95 (0.90 – 1.00)*	0.96 (0.90 – 1.01)		
Absolute FVC (100 ml increments)			0.99 (0.94 – 1.04)	0.98 (0.94 – 1.03)
Bronchiectasis severity score (3 point increments, 0-6) §	0.84 (0.61 – 1.15)	0.92 (0.66 – 1.27)	0.86 (0.62 – 1.18)	0.93 (0.67 – 1.29)
Abnormal parenchyma (10% increments) ‖	1.10 (0.91 – 1.33)	1.11 (0.92 – 1.34)	1.15 (0.96 – 1.39)	1.15 (0.96 – 1.39)

*OR statistically significant at p<0.05 level.

† Cough, breathlessness, sputum production, or wheeze with frequency ≥few days/month.

‡ Participants excluded if absent HIV status at TB treatment completion (n=2), no valid baseline spirometry (n=36), no baseline CT imaging (n=20).

§ Bronchiectasis severity score (0-18): unweighted sum of 6x lobar bronchiectasis scores ranging from 0 (no bronchiectasis) to 3 (severe dilatation >3 times diameter of adjacent vessel), such that increment of 3 points represents one additional lobe with severe airway dilatation, or combination of 1-3 lobes with less severe disease.

‖Total % of parenchymal abnormalities across lung, including: parenchymal banding, atelectasis, consolidation, ground glass change, emphysema, emphysematoid destruction, cavities / cystic airspaces. Excluding mosaicism which represents airway/vascular rather than lung tissue abnormality.

Meghji J, *et al. Thorax* 2020; 75:1–10. doi: 10.1136/thoraxjnl-2019-213808

Appendix 12: Factors predicting acute respiratory events over 1-year, including sensitivity analyses

Table E13: Logistic regression models of baseline parameters associated with the presence of any acute respiratory events[†] over 1-year follow up (n=335)[‡].

Variables measured at TB treatment end	Univariate OR (95% CI)	Multivariate, controlling for baseline FEV_1 [§] OR (95% CI)	Multivariate, controlling for baseline FVC [§] OR (95% CI)
Age	1.01 (0.99 – 1.04)	1.01 (0.98 – 1.04)	1.02 (0.98 -1.05)
Female sex	1.29 (0.71 – 2.36)	0.43 (0.16 – 1.18)	0.43 (0.15 – 1.19)
Height	0.96 (0.92 – 0.99)*	0.96 (0.91 – 1.01)	0.96 (0.91 – 1.02)
HIV status<br>- Negative<br>- Positive, CD4≥200<br>- Positive, CD4<200	 1.0<br>0.51 (0.26 – 0.996)*<br>0.50 (0.23 – 1.06)	 1.0<br>0.43 (0.20 – 0.90)*<br>0.34 (0.14 – 0.85)*	 1.0<br>0.42 (0.20 – 0.89)*<br>0.33 (0.13 – 0.82)*
Microbiologically proven TB	1.05 (0.52 – 2.11)	1.21 (0.56 – 2.63)	1.23 (0.56 – 2.68)
BMI (kg/m2)	0.98 (0.89 – 1.09)	1.03 (0.91 – 1.15)	1.03 (0.92 – 1.15)
Hb (g/dL)	0.90 (0.78 – 1.04)	0.86 (0.71 – 1.04)	0.86 (0.71 – 1.04)
Pack-year smoking history	0.98 (0.91 – 1.05)	0.98 (0.91 – 1.05)	0.97 (0.91 – 1.05)
Maximum education ≤ 1ry school	1.02 (0.57 – 1.84)	0.81 (0.41 – 1.61)	0.87 (0.44 – 1.70)
Respiratory symptoms ≥monthly	3.00 (1.49 – 6.04)*	2.60 (1.25 – 5.42)*	2.60 (1.25 – 5.42)*
Absolute FEV1 (100 ml increments)	0.94 (0.89 – 0.98)*	0.94 (0.88 – 1.01)	
Absolute FVC (100 ml increments)	0.95 (0.91 – 0.99)*		0.95 (0.89 – 1.02)
Bronchiectasis severity score (3-point increments, 0-6)[‖]	0.94 (0.70 – 1.27)	0.81 (0.54 – 1.22)	0.80 (0.52 – 1.21)
Abnormal parenchyma (10% increments) [††]	1.05 (0.90 – 1.23)	0.99 (0.79 – 1.25)	1.01 (0.80 – 1.27)

*OR statistically significant at p<0.05 level.

[†]Present if ≥1 acute respiratory event (an unscheduled visit to health care provider (outpatient or inpatient) due to a respiratory complaint (cough, breathlessness, sputum, wheeze, chest pain)) during 6- or 12-month study follow-up.

[‡]Participants excluded if absent HIV status at TB treatment completion (n=2), no valid baseline spirometry (n=36), no baseline HRCT imaging (n=20), or no outcome data obtained over follow-up period (n=12). Includes participants contributing either 6-month (n=13) and 12-month (n=322) follow-up.

[§]Co-linearity of FEV_1 and FVC precludes inclusion of both in a single model – separate models constructed to control for baseline level of each.

[‖]Bronchiectasis severity score (0-18): unweighted sum of 6x lobar bronchiectasis scores ranging from 0 (no bronchiectasis) to 3 (severe dilatation >3 times diameter of adjacent vessel), such that increment of 3 points represents one additional lobe with severe airway dilatation, or combination of 1-3 lobes with less severe disease.

[††]Total % of parenchymal abnormalities across lung, including: parenchymal banding, atelectasis, consolidation, ground glass change, emphysema, emphysematoid destruction, cavities / cystic airspaces. Excluding mosaicism which represents airway/vascular rather than lung tissue abnormality.

Meghji J, *et al. Thorax* 2020; 75:1–10. doi: 10.1136/thoraxjnl-2019-213808

Table E14: Sensitivity analyses of logistic regression models of baseline parameters associated with the presence of any acute respiratory events† over 1-year follow up‡, with outcomes for those lost to follow-up allocated as positive or negative.

Analysis 1: Participants with 6-months follow-up only, who were not known to have already had a respiratory event, assumed to have had a respiratory event after LTFU (n=335).

Analysis 2: Participants with 6-months follow-up only who were not known to have already had a respiratory event assumed to have had a respiratory event after LTFU, AND those with no follow-up assumed to have had a respiratory event (n=347).

Analysis 3: Participants with no-follow up assumed to have had a respiratory event (n=347).

Variables measured at TB treatment end	Multivariate model, with FEV_1			Multivariate model, with FVC		
	Analyses 1	Analysis 2	Analysis 3	Analyses 1	Analysis 2	Analysis 3
Age	1.00 (0.97 – 1.04)	1.00 (0.97 – 1.03)	1.01 (0.98 – 1.05)	1.01 (0.98 – 1.04)	1.01 (0.98 – 1.04)	1.02 (0.99 – 1.05)
Female sex	0.42 (0.16 – 1.09)	0.60 (0.25 – 1.46)	0.42 (0.16 – 1.15)	0.46 (0.17 – 1.19)	0.65 (0.27 – 1.59)	0.41 (0.15 – 1.15)
Height	0.96 (0.91 – 1.01)	0.99 (0.95 – 1.04)	0.95 (0.90 -1.01)	0.96 (0.91 – 1.01)	0.99 (0.94 – 1.03)	0.96 (0.90 – 1.01)
HIV status<br>- Negative<br>- Positive, CD4≥200<br>- Positive, CD4<200	1.0<br>0.39 (0.19 – 0.80)*<br>0.40 (0.17 – 0.92)*	1.0<br>0.36 (0.18 – 0.72)*<br>0.50 (0.24 – 1.07)	1.0<br>0.44 (0.21 – 0.93)*<br>0.34 (0.14 – 0.84)*	1.0<br>0.39 (0.19 – 0.79)*<br>0.38 (0.17 – 0.88)*	1.0<br>0.36 (0.18 – 0.71)*<br>0.49 (0.23 – 1.03)*	1.0<br>0.44 (0.21 – 0.92)*<br>0.33 (0.13 – 0.81)*
Microbiologically proven TB	1.44 (0.68 – 3.02)	1.47 (0.73 – 2.94)	1.21 (0.56 – 2.62)	1.43 (0.68 – 3.01)	1.46 (0.73 – 2.93)	1.21 (0.56 – 2.64)
BMI (kg/m2)	1.06 (0.95 – 1.17)	1.03 (0.94 – 1.15)	1.03 (0.91 – 1.15)	1.05 (0.95 – 1.17)	1.04 (0.94 – 1.15)	1.03 (0.92 – 1.15)
Hb (g/dL)	0.84 (0.70 – 1.00)*	0.84 (0.71 – 1.00)*	0.87 (0.72 – 1.05)	0.84 (0.70 – 1.00)	0.84 (0.71 – 1.00)*	0.87 (0.72 – 1.05)
Pack-year smoking history	0.96 (0.89 – 1.04)*	0.95 (0.88 – 1.03)	0.98 (0.91 – 1.05)	0.96 (0.89 – 1.04)	0.95 (0.88 – 1.03)	0.98 (0.91 – 1.05)
Maximum education ≤ 1ry school	0.83 (0.44 – 1.58)	0.78 (0.43 – 1.42)	0.82 (0.41 – 1.61)	0.87 (0.46 – 1.63)	0.81 (0.45 – 1.47)	0.87 (0.44 – 1.69)
Respiratory symptoms ≥monthly	2.33 (1.21– 4.52)*	2.49 (1.34 – 4.63)*	2.45 (1.18 – 5.10)*	2.36 (1.22 – 4.56)*	2.52 (1.35 – 4.68)*	2.46 (1.18 – 5.12)*
Absolute FEV1 (100 ml increments)	0.96 (0.90 – 1.03)	0.96 (0.91 – 1.02)	0.95 (0.89 – 1.02)			
Absolute FVC (100 ml increments)				0.98 (0.93 – 1.04)	0.98 (0.93 – 1.04)	0.96 (0.90 – 1.02)
Bronchiectasis severity score (3 point increments, 0-6) §	0.75 (0.50 – 1.12)	0.77 (0.53 – 1.11)	0.82 (0.54 – 1.23)	0.75 (0.50 – 1.13)	0.77 (0.53 – 1.12)	0.80 (0.52 – 1.22)
Abnormal parenchyma (10% increments) ‖	1.00 (0.80 – 1.25)	1.01 (0.82 – 1.25)	0.99 (0.79 – 1.25)	1.03 (0.83 - 1.28)	1.04 (0.85 – 1.28)	1.01 (0.80 – 1.27)

*OR statistically significant at p<0.05 level.

†Present if ≥1 acute respiratory event (an unscheduled visit to health care provider (outpatient or inpatient) due to a respiratory complaint (cough, breathlessness, sputum, wheeze, chest pain)) documented in health passport +/- self reported during 6- or 12-month study follow-up.

‡Participants excluded if absent HIV status at TB treatment completion (n=2), no valid baseline spirometry (n=36), no baseline CT imaging (n=20), or no outcome data obtained over follow-up period (n=12). Includes participants contributing either 6-month (n=13) and 12-month (n=322) follow-up.

[§] Bronchiectasis severity score (0-18): unweighted sum of 6x lobar bronchiectasis scores ranging from 0 (no bronchiectasis) to 3 (severe dilatation >3 times diameter of adjacent vessel), such that increment of 3 points represents one additional lobe with severe airway dilatation, or combination of 1-3 lobes with less severe disease.
[‖] Total % of parenchymal abnormalities across lung, including: parenchymal banding, atelectasis, consolidation, ground glass change, emphysema, emphysematoid destruction, cavities / cystic airspaces. Excluding mosaicism which represents airway/vascular rather than lung tissue abnormality.

Appendix 13: Factors predicting spirometry at 1-year

Table E15: Linear regression model, to determine the effect of any respiratory events in the 1-year follow up period, on spirometry parameters 1-year following TB treatment completion (n=296)†.
All univariate & multivariate models coefficients include adjustment for participant age (yrs), sex, and height (cm).

Variables	Univariate (ml, 95% CI)	Multivariate, partial model (ml, 95% CI)	Multivariate, full model (ml, 95% CI)
Absolute FEV_1 (ml) at 1-year			
HIV positive status	192.30 (69.39 – 315.21)*	173.89 (52.57 – 295.20)*	85.04 (35.68 – 134.40)
Microbiologically proven TB	-81.57 (-222.21 – 59.07)	-32.16 (-168.75 – 104.44)	-33.36 (-88.38 – 21.65)
BMI (kg/m2)	41.10 (19.72 – 62.48)*	37.83 (16.79 – 58.88)*	0.93 (-7.95 – 9.81)
Pack-year smoking history	-11.74 (-24.12 – 0.63)	-8.27 (-20.35 – 3.80)	-2.82 (-7.65 – 2.02)
Maximum education ≤ 1ry school	-138.51 (-265.89 - -11.14)*	-128.39 (-253.25 - -3.52)*	-8.25 (-58.89 – 42.38)
Respiratory symptoms ≥monthly	-181.70 (-300.56 - -62.84)*		-9.42 (-57.04 – 38.20)
Absolute FEV1 at baseline (100 ml increments)	87.80 (83.47 – 92.13)*		86.74 (81.62 – 91.86)*
Bronchiectasis severity score (3-point increments, 0-6)	-194.40 (-248.03 - -140.77)*		-5.32 (-35.04 – 24.40)
Abnormal parenchyma (10% increments)	-151.35 (-181.37 - -121.34)*		6.45 (-12.62 – 25.52)
≥1 acute respiratory event during follow-up	-202.91 (-366.66 - -39.17)*		-81.97 (-146.95 - -17.00)*
Absolute FVC (ml) at 1-year			
HIV positive status	181.00 (53.19 – 308.81)*	165.79 (38.00 – 293.58)*	108.55 (55.12 – 161.99)*
Microbiologically proven TB	-1.24 (-147.40 – 144.92)	37.50 (-106.38 – 181.38)	5.49 (-54.15 – 65.13)
BMI (kg/m2)	39.17 (16.92 – 61.42)	36.91 (14.75 – 59.08)*	-4.06 (-13.71 – 5.59)
Pack-year smoking history	-9.43 (-22.29 – 3.43)	-7.72 (-20.44 – 5.00)	-1.35 (-6.59 – 3.89)
Maximum education ≤ 1ry school	-24.09 (-157.17 – 109.00)	-19.29 (-150.82 – 112.24)	22.37 (-32.24 – 76.98)
Respiratory symptoms ≥monthly	-185.13 (-308.45 - -61.82)*		-7.55 (-59.16 – 44.05)
Absolute FVC at baseline (100 ml increments)	84.50 (80.01 – 88.99)*		85.73 (80.71 – 90.74)*
Bronchiectasis severity score (3-point increments, 0-6)	-178.93 (-235.56 - -122.31)*		-14.22 (-46.52 – 18.08)
Abnormal parenchyma (10% increments)	-127.27 (-160.15 - -94.39)*		20.95 (0.94 – 40.97)*
≥1 acute respiratory event during follow-up	-256.72 (-425.66 - -87.77)*		-121.78 (-192.19 - -51.37)*

*Co-efficients statistically significant at p<0.05 level.

†Participants excluded if absent HIV status at TB treatment completion (n=2), no valid baseline spirometry (n=36), no baseline HRCT imaging (n=20), no valid 12-month spirometry (n=48), or no data on events obtained over follow-up period (n=12).

References

1. National Institute for Occupational Safety & Health. Spirometry quality assurance: Common errors and their impact on test results. Atlanta, USA: Department for Health & Human Services, Centres for Disease Control & Prevention; 2012.

2. Burden of Obstructive Lung Disease Study. Spirometry quality control guidelines. 2009.

3. Quanjer PH, Stanojevic S, Cole TJ, Baur X, Hall GL, Culver BH, et al. Multi-ethnic reference values for spirometry for the 3-95-yr age range: the global lung function 2012 equations. Eur Respir J. 2012;40(6):1324-43.

4. Pellegrino R, Viegi G, Brusasco V, Crapo RO, Burgos F, Casaburi R, et al. Interpretative strategies for lung function tests. Eur Respir J. 2005;26(5):948-68.

5. Meghji J, Simpson H, Squire SB, Mortimer K. A Systematic Review of the Prevalence and Pattern of Imaging Defined Post-TB Lung Disease. PLoS One. 2016;11(8):e0161176.

6. Hansell DM, Bankier AA, MacMahon H, McLoud TC, Muller NL, Remy J. Fleischner Society: Glossary of Terms for Thoracic Imaging. Radiology. 2008;246:697 - 722.

7. Long R, Maycher B, Dhar A, Manfreda J, Hershfield E, Anthonisen N. Pulmonary tuberculosis treated with directly observed therapy: serial changes in lung structure and function. Chest. 1998;113(4):933-43.

8. Sin DD, Anthonisen NR, Soriano JB, Agusti AG. Mortality in COPD: Role of comorbidities. Eur Respir J. 2006;28(6):1245-57.

9. Burney P, Jithoo A, Kato B, Janson C, Mannino D, Nizankowska-Mogilnicka E, et al. Chronic obstructive pulmonary disease mortality and prevalence: the associations with smoking and poverty--a BOLD analysis. Thorax. 2014;69(5):465-73.

10. Burney PG, Hooper R. Forced vital capacity, airway obstruction and survival in a general population sample from the USA. Thorax. 2011;66(1):49-54.

11. Chalmers JD, Goeminne P, Aliberti S, McDonnell MJ, Lonni S, Davidson J, et al. The bronchiectasis severity index: An international derivation and validation study. Am J Respir Crit Care Med. 2014;189(5):576-85.

12. Martinez-Garcia MA, de Gracia J, Vendrell Relat M, Giron RM, Maiz Carro L, de la Rosa Carrillo D, et al. Multidimensional approach to non-cystic fibrosis bronchiectasis: the FACED score. Eur Respir J. 2014;43(5):1357-67.

13. Boutou AK, Nair A, Douraghi-Zadeh D, Sandhu R, Hansell DM, Wells AU, et al. A combined pulmonary function and emphysema score prognostic index for staging in Chronic Obstructive Pulmonary Disease. PLoS One. 2014;9(10):e111109.

14. Goh NS, Desai SR, Veeraraghavan S, Hansell DM, Copley SJ, Maher TM, et al. Interstitial lung disease in systemic sclerosis: a simple staging system. Am J Respir Crit Care Med. 2008;177(11):1248-54.

15. Meghji J, Nadeau G, Davis KJ, Wang D, Nyirenda MJ, Gordon SB, et al. Non-communicable Lung Disease in Sub Saharan Africa: a Community-based Cross-sectional Study of Adults in Urban Malawi. Am J Respir Crit Care Med. 2016;194(1):67-76.

Meghji J, *et al. Thorax* 2020; 75:1–10. doi: 10.1136/thoraxjnl-2019-213808
